# Appraising the role of previously reported risk factors in epithelial ovarian cancer risk: A Mendelian randomization analysis

**DOI:** 10.1371/journal.pmed.1002893

**Published:** 2019-08-07

**Authors:** James Yarmolinsky, Caroline L. Relton, Artitaya Lophatananon, Kenneth Muir, Usha Menon, Aleksandra Gentry-Maharaj, Axel Walther, Jie Zheng, Peter Fasching, Wei Zheng, Woo Yin Ling, Sue K. Park, Byoung-Gie Kim, Ji-Yeob Choi, Boyoung Park, George Davey Smith, Richard M. Martin, Sarah J. Lewis

**Affiliations:** 1 MRC Integrative Epidemiology Unit, University of Bristol, Bristol, United Kingdom; 2 Population Health Sciences, Bristol Medical School, University of Bristol, Bristol, United Kingdom; 3 National Institute for Health Research Bristol Biomedical Research Centre, University of Bristol and University Hospitals Bristol NHS Foundation Trust, Bristol, United Kingdom; 4 Division of Population Health, Health Services Research and Primary Care, School of Health Sciences, Faculty of Biology, Medicine and Health, University of Manchester, Manchester, United Kingdom; 5 MRC Clinical Trials Unit, Institute for Clinical Trials and Methodology, University College London, London, United Kingdom; 6 Bristol Cancer Institute, University Hospitals Bristol NHS Foundation Trust, Bristol, United Kingdom; 7 Department of Gynecology and Obstetrics, University Hospital Erlangen, Comprehensive Cancer Center Erlangen–EMN, Friedrich-Alexander University Erlangen–Nuremberg, Erlangen, Germany; 8 Division of Epidemiology, Vanderbilt University Medical Center, Vanderbilt University, Nashville, Tennessee, United States of America; 9 Faculty of Medicine, University of Malaya, Kuala Lumpur, Malaysia; 10 Department of Preventive Medicine, Seoul National University College of Medicine, Seoul, South Korea; 11 Cancer Research Institute, Seoul National University, Seoul, South Korea; 12 Department of Biomedical Science, Seoul National University Graduate School, Seoul, South Korea; 13 Department of Obstetrics and Gynecology, Samsung Medical Center, Sungkyunkwan University School of Medicine, Seoul, South Korea; 14 Department of Medicine, College of Medicine, Hanyang University, Seoul, South Korea; Imperial College London, UNITED KINGDOM

## Abstract

**Background:**

Various risk factors have been associated with epithelial ovarian cancer risk in observational epidemiological studies. However, the causal nature of the risk factors reported, and thus their suitability as effective intervention targets, is unclear given the susceptibility of conventional observational designs to residual confounding and reverse causation. Mendelian randomization (MR) uses genetic variants as proxies for risk factors to strengthen causal inference in observational studies. We used MR to evaluate the association of 12 previously reported risk factors (reproductive, anthropometric, clinical, lifestyle, and molecular factors) with risk of invasive epithelial ovarian cancer, invasive epithelial ovarian cancer histotypes, and low malignant potential tumours.

**Methods and findings:**

Genetic instruments to proxy 12 risk factors were constructed by identifying single nucleotide polymorphisms (SNPs) that were robustly (*P* < 5 × 10^−8^) and independently associated with each respective risk factor in previously reported genome-wide association studies. These risk factors included genetic liability to 3 factors (endometriosis, polycystic ovary syndrome, type 2 diabetes) scaled to reflect a 50% higher odds liability to disease. We obtained summary statistics for the association of these SNPs with risk of overall and histotype-specific invasive epithelial ovarian cancer (22,406 cases; 40,941 controls) and low malignant potential tumours (3,103 cases; 40,941 controls) from the Ovarian Cancer Association Consortium (OCAC). The OCAC dataset comprises 63 genotyping project/case–control sets with participants of European ancestry recruited from 14 countries (US, Australia, Belarus, Germany, Belgium, Denmark, Finland, Norway, Canada, Poland, UK, Spain, Netherlands, and Sweden). SNPs were combined into multi-allelic inverse-variance-weighted fixed or random effects models to generate effect estimates and 95% confidence intervals (CIs). Three complementary sensitivity analyses were performed to examine violations of MR assumptions: MR–Egger regression and weighted median and mode estimators. A Bonferroni-corrected *P* value threshold was used to establish strong evidence (*P* < 0.0042) and suggestive evidence (0.0042 < *P* < 0.05) for associations. In MR analyses, there was strong or suggestive evidence that 2 of the 12 risk factors were associated with invasive epithelial ovarian cancer and 8 of the 12 were associated with 1 or more invasive epithelial ovarian cancer histotypes. There was strong evidence that genetic liability to endometriosis was associated with an increased risk of invasive epithelial ovarian cancer (odds ratio [OR] per 50% higher odds liability: 1.10, 95% CI 1.06–1.15; *P* = 6.94 × 10^−7^) and suggestive evidence that lifetime smoking exposure was associated with an increased risk of invasive epithelial ovarian cancer (OR per unit increase in smoking score: 1.36, 95% CI 1.04–1.78; *P* = 0.02). In analyses examining histotypes and low malignant potential tumours, the strongest associations found were between height and clear cell carcinoma (OR per SD increase: 1.36, 95% CI 1.15–1.61; *P* = 0.0003); age at natural menopause and endometrioid carcinoma (OR per year later onset: 1.09, 95% CI 1.02–1.16; *P* = 0.007); and genetic liability to polycystic ovary syndrome and endometrioid carcinoma (OR per 50% higher odds liability: 0.89, 95% CI 0.82–0.96; *P* = 0.002). There was little evidence for an association of genetic liability to type 2 diabetes, parity, or circulating levels of 25-hydroxyvitamin D and sex hormone binding globulin with ovarian cancer or its subtypes. The primary limitations of this analysis include the modest statistical power for analyses of risk factors in relation to some less common ovarian cancer histotypes (low grade serous, mucinous, and clear cell carcinomas), the inability to directly examine the association of some ovarian cancer risk factors that did not have robust genetic variants available to serve as proxies (e.g., oral contraceptive use, hormone replacement therapy), and the assumption of linear relationships between risk factors and ovarian cancer risk.

**Conclusions:**

Our comprehensive examination of possible aetiological drivers of ovarian carcinogenesis using germline genetic variants to proxy risk factors supports a role for few of these factors in invasive epithelial ovarian cancer overall and suggests distinct aetiologies across histotypes. The identification of novel risk factors remains an important priority for the prevention of epithelial ovarian cancer.

## Abstract 2

James Yarmolinsky and colleagues assess risk factors for epithelial ovarian cancer using a genetic approach.

## Author summary

**Why was this study done?:**

- Numerous reproductive, lifestyle, and molecular factors have been linked to risk of epithelial ovarian cancer in observational epidemiological studies. It is unclear whether these associations represent causal relationships or merely reflect residual confounding, reverse causation, or other forms of bias inherent to conventional epidemiological designs.
- Mendelian randomization (MR) uses germline genetic variants as proxies for risk factors to generate more reliable evidence of the causal effect of risk factors on disease-related outcomes.

**What did the researchers do and find?:**

- We employed MR analysis to systematically evaluate the association of 12 previously reported risk factors with risk of invasive epithelial ovarian cancer, invasive epithelial ovarian cancer histotypes, and low malignant potential tumours in up to 25,509 cases and 40,941 controls in the Ovarian Cancer Association Consortium.
- In MR analysis, only 2 of 12 previously reported risk factors (genetic liability to endometriosis and lifetime smoking exposure) were associated with invasive epithelial ovarian cancer risk.
- When analyses were stratified on invasive epithelial ovarian cancer histotypes and low malignant potential tumours, 8 risk factors were associated with 1 or more epithelial ovarian cancer subtypes. The strongest associations identified were positive associations of height with clear cell carcinoma and age at natural menopause with endometrioid carcinoma and an inverse association of genetic liability to polycystic ovary syndrome with endometrioid carcinoma.

**What do these findings mean?:**

- Beyond implicating genetic liability to endometriosis and lifetime smoking exposure in invasive epithelial ovarian cancer risk, these analyses found little evidence to support roles for several previously reported risk factors for this malignancy in disease aetiology.
- Our findings support distinct risk profiles across invasive epithelial ovarian cancer histotypes, which could have important implications for studies of disease prognosis, but which complicate the design of effective prevention strategies targeting ovarian cancer as a whole.

## Introduction

Ovarian cancer is the second most common gynaecological cancer in the US and Western Europe and accounts for more deaths than all other gynaecological cancers combined [1,2]. The prognosis for ovarian cancer is generally poor because women typically present with advanced disease due to the non-specific nature of symptoms and because of the lack of established screening tests [3–5]. Given the limited success of secondary prevention strategies and the sporadic nature of 90% of cases, primary prevention of ovarian cancer may serve as an important vehicle for disease control [6]. However, few risk factors have consistently been linked to epithelial ovarian cancer, which accounts for 85%–90% of ovarian cancers, in observational epidemiological studies, and most previous studies have failed to stratify analyses across clinically distinct histotypes [7–10]. Stratification is necessary because of the previously reported heterogeneity in risk factor–histotype associations in epidemiological analyses [8–10]. Further, the causal nature of the risk factors reported, and thus their suitability as effective intervention targets, is unclear given the susceptibility of conventional observational designs to residual confounding and reverse causation.

Mendelian randomization (MR) is an analytical approach that uses germline genetic variants as instruments (“proxies”) for risk factors, to examine the causal effects of these factors on disease outcomes in observational settings [11,12]. Since germline genetic variants are randomly assorted at meiosis, MR analyses should be less prone to confounding by lifestyle and environmental factors than conventional observational studies. Further, since germline genetic variants are fixed at conception and cannot be influenced by subsequent disease processes, MR analyses are not subject to reverse causation bias. An additional advantage of MR is that it can be implemented using summary genetic association data from 2 independent samples representing (1) the genetic variant–risk factor associations and (2) the genetic variant–outcome associations (known as the 2-sample MR approach). This approach provides an efficient and statistically robust method of appraising causal relationships between risk factors and disease outcomes. To date, MR analyses of ovarian cancer have reported a positive association of body mass index (BMI) with non–high grade serous carcinoma (HGSC) but a null association with HGSC [13], a positive association of height with ovarian cancer [14], and an inverse association of 25-hydroxyvitamin D with ovarian cancer, including HGSC [15]. However, these analyses were limited by modest sample sizes (10,065–16,395 cases), restricting power for subtype-stratified analysis.

Given the unclear causal relevance of previously reported observational associations of risk factors in the aetiology of epithelial ovarian cancer, and limited exploration of their causality in MR analyses to date, a 2-sample MR analysis was performed to evaluate the association of 12 previously reported factors with risk of invasive epithelial ovarian cancer, invasive epithelial ovarian cancer histotypes, and low malignant potential tumours.

## Methods

### Ovarian cancer population

Summary genetic association data were obtained on 25,509 women with epithelial ovarian cancer and 40,941 controls of European descent. These women had been genotyped using the Illumina Custom Infinium array (OncoArray) as part of the Ovarian Cancer Association Consortium (OCAC) genome-wide association study (GWAS) [16,17]. This dataset comprises 63 genotyping project/case–control sets representing participants of European ancestry recruited from 14 countries (US, Australia, Belarus, Germany, Belgium, Denmark, Finland, Norway, Canada, Poland, UK, Spain, Netherlands, and Sweden), with some studies contributing samples to more than 1 genotyping project and some case–control sets representing a combination of multiple individual studies. Genotype data were obtained either by direct genotyping using an Illumina Custom Infinium array (OncoArray) consisting of approximately 530,000 SNPs or by imputation with reference to the 1000 Genomes Project Phase 3 dataset [18]. The data included 22,406 invasive epithelial ovarian cancer cases (40,941 controls) involving the following invasive epithelial ovarian cancer histotypes: HGSC (*n* = 13,037), low grade serous carcinoma (*n* = 1,012), mucinous carcinoma (*n* = 1,417), endometrioid carcinoma (*n* = 2,810), and clear cell carcinoma (*n* = 1,366). Invasive histotypes classified as “other” by OCAC (*n* = 2,764 cases) were included in analyses of overall invasive epithelial ovarian cancer but were not assessed separately. Analyses were also performed for low malignant potential tumours (*n* = 3,103), which included 1,954 serous and 1,149 mucinous tumours. Ethical approval from relevant research ethics committees was obtained for all studies in OCAC, and written, informed consent was obtained from all participants in these studies. Further details about the OCAC study and OncoArray analyses are available in S1 Text.

### Identification of previously reported risk factors and instrument selection

Previously reported risk factors for epithelial ovarian cancer were identified from a literature review, using PubMed and Web of Science, of narrative and systematic review articles summarising findings from observational epidemiological studies [19–24] and through consultation with the Cancer Research UK website (https://www.cancerresearchuk.org/about-cancer/ovarian-cancer/risks-causes) and the World Cancer Research Fund/American Institute for Cancer Research (WCRF/AICR) *Ovarian Cancer 2014 Report* [25]. Genetic instruments were then identified for these risk factors by consulting the preprint server bioRxiv (http://www.biorxiv.org/) and 2 catalogues of summary GWAS data: the NHGRI-EBI (National Human Genome Research Institute–European Bioinformatics Institute) GWAS catalogue and MR-Base [26,27]. The complete PubMed and Web of Science search strategies and instrument selection criteria are presented in S1 and S2 Texts, respectively.

**Fig 1:**
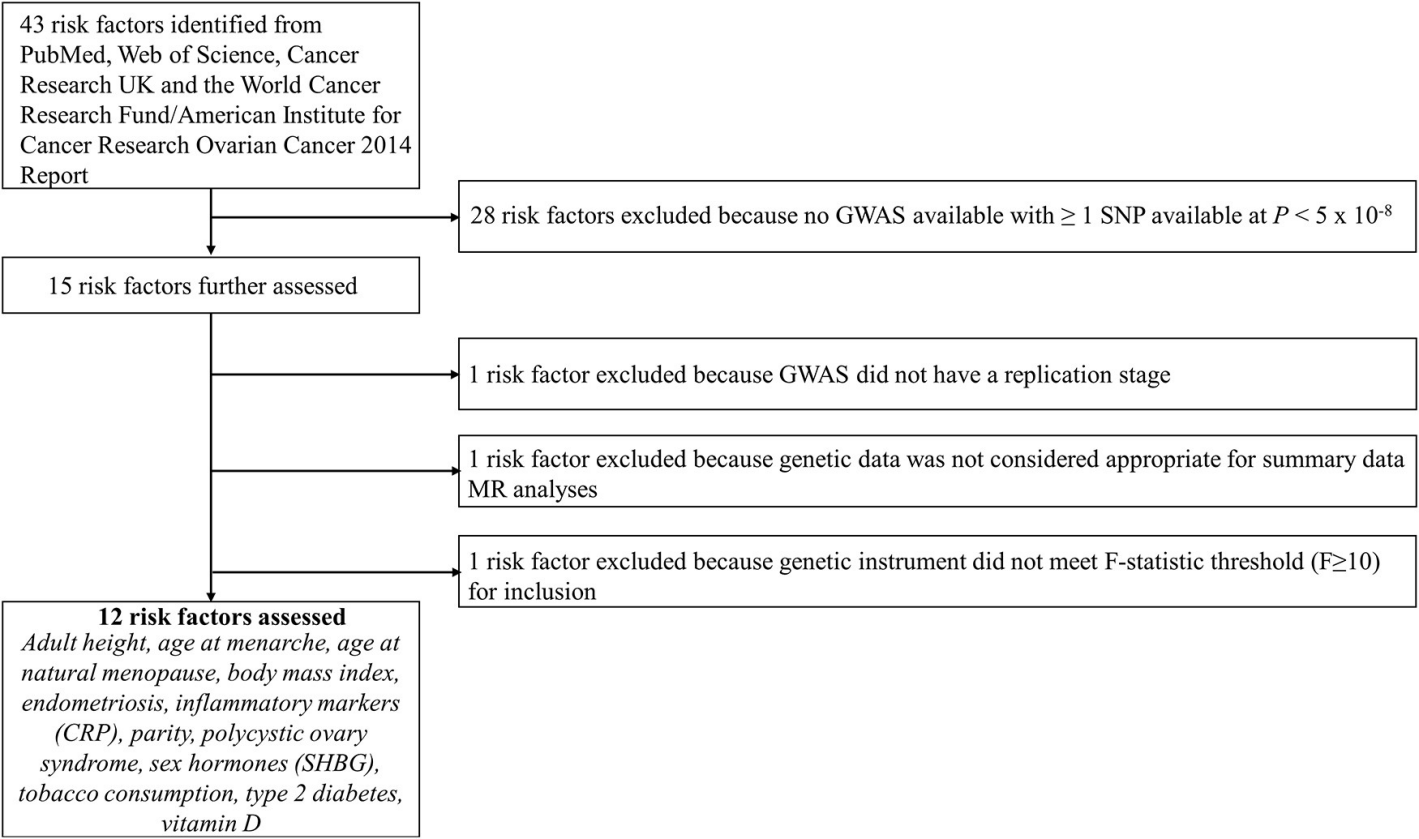
Flowchart for risk factor inclusion. CRP, C-reactive protein; GWAS, genome-wide association study; MR, Mendelian randomization; SHBG, sex hormone binding globulin; SNP, single nucleotide polymorphism.

In total, 12 risk factors with a suitable genetic instrument were included in the analysis: 3 reproductive factors (age at menarche, age at natural menopause, and parity) [28–31], 2 anthropometric traits (BMI and height) [32,33], 3 clinical factors (genetic liabilities to type 2 diabetes, endometriosis, and polycystic ovary syndrome [PCOS]) [34–36], 1 lifestyle factor (lifetime smoking exposure) [37,38], and 3 molecular risk factors (circulating 25-hydroxyvitamin D, C-reactive protein [CRP], and sex hormone binding globulin) [39,40]. Lifetime smoking exposure is a composite score that captures smoking duration, heaviness, and cessation among both smokers and non-smokers. A step-by-step overview of risk factor inclusion along with a flow chart of these processes and a list of all risk factors ascertained for inclusion are presented in Fig 1 and S1 Text.

### Statistical analyses

The use of genetic instruments for exposures in an MR framework allows for unbiased causal effects of risk factors on disease outcomes to be estimated if the following assumptions are met: (1) the genetic instrument (typically, 1 or more independent SNPs) is robustly associated with the risk factor of interest; (2) the instrument is not associated with any confounding factor(s) of the association between the risk factor and outcome; and (3) there is no pathway through which the instrument influences the outcome except through the risk factor (“exclusion restriction criterion”).

After obtaining effect estimates from relevant GWASs, SNPs were pruned for linkage disequilibrium at *R*^2^ < 0.001 at a clumping distance of 10,000 kilobases from the lead SNP at *P* < 5 × 10^−8^ with reference to the 1000 Genomes Project (http://www.internationalgenome.org/). SNP–risk factor association estimates were derived from either sex-combined or female-specific analyses as presented in Table 1, and methodological considerations for when instruments were derived from sex-combined versus female-specific analyses are presented in S2 Text.

**Table 1:** Catalogue of GWASs used for genetic instruments and estimates of instrument strength for previously reported risk factors.

Reported risk factor	GWAS	Sex	Number of SNPs available^1^	Number of SNPs used^2^	*R*^2^	*F*-statistic
**Reproductive factors**						
Age at menarche	Day et al. [30]	Women	368	329	5.1	55
Age at natural menopause	Day et al. [29]	Women	42	35	4.2	86
Parity	Barban et al. [28]	Combined	2	2	0.02	33
**Anthropometric traits**						
Body mass index	Locke et al. [32]	Combined	77	66	2.2	110
Height	Wood et al. [33]	Combined	386	345	11.8	98
**Clinical factors**						
Endometriosis	Sapkota et al. [36]	Women	10	10	0.24	49
Polycystic ovary syndrome	Day et al. [34]	Women	14	11	0.35	36
Type 2 diabetes	Morris et al. [35]	Combined	10	10	0.34	24
**Lifestyle factors**						
Lifetime smoking exposure	Wootton et al. [38]	Combined	124	115	1.2	49
**Molecular risk factors**						
C-reactive protein	Dehghan et al. [40]	Combined	9	8	3.6	382
SHBG	Coviello et al. [39]	Combined	9	8	4.3	121
25-hydroxyvitamin D	Jiang et al. [37]	Combined	6	5	2.6	423

*R*^2^ represents the proportion of variance in a risk factor explained by the genetic instrument. *F*-statistic represents the strength of the association between the genetic instrument and levels of the risk factor.

^1^Corresponds to the number of SNPs available after pruning top SNPs reported in corresponding linkage disequilibrium at *R*^2^ < 0.001 at a clumping distance of 10,000 kilobases from the lead SNP at *P* < 5 × 10^−8^.

^2^Corresponds to the number of SNPs (or linkage disequilibrium proxies) available in ovarian cancer datasets.

GWAS, genome-wide association study; SHBG, sex hormone binding globulin; SNP, single nucleotide polymorphism.

Estimates of the proportion of variance in each risk factor explained by the genetic instruments (*R*^2^) and the strength of the association between the genetic instruments and risk factors (*F*-statistics) were generated using methods previously described [41]. F-statistics can be used to examine whether results are likely to be influenced by weak instrument bias, i.e., reduced statistical power to reject the null hypothesis when an instrument explains a limited proportion of the variance in a risk factor. We excluded risk factors with an *F*-statistic of <10 in order to minimise weak instrument bias.

For risk factors with 2 or 3 SNPs as instruments, inverse-variance-weighted (IVW) fixed effects models were used to generate effect estimates, and for risk factors with greater than 3 SNPs, IVW multiplicative random effects models (allowing overdispersion in the model) were used [42]. The combination of multiple SNPs into a multi-allelic instrument increases the proportion of variance in a risk factor explained by the instrument. Effect estimates from these models represent a weighted average of individual Wald ratios across SNPs using IVW meta-analysis. Effect estimates for associations of genetic liability to binary clinical factors (endometriosis, PCOS, type 2 diabetes) with ovarian cancer outcomes represent the effect of a 50% higher odds liability to these clinical factors (i.e., equivalent to the effect of a 1.5-fold increase in the odds liability to disease). These estimates were obtained by scaling the natural log odds ratio (OR) of ovarian cancer outcome per natural log OR of clinical factor by a factor of 1.5. As these analyses were not performed in clinical samples (i.e., were not restricted to individuals diagnosed with the disease states examined), the estimates represent subdiagnostic traits or pathophysiological pathways leading to susceptibility to disease, rather than the disease itself. To account for multiple testing across analyses, a Bonferroni correction was used to establish *P* value thresholds for strong evidence (*P* < 0.0042) (false positive rate = 0.05/12 risk factors) and suggestive evidence (0.0042 < *P* < 0.05) for reported associations.

When using genetic instruments, there is potential for horizontal pleiotropy—when a genetic variant has an effect on 2 or more traits through independent biological pathways, a violation of the third instrumental variable assumption. This was examined by performing 3 complementary sensitivity analyses, each of which makes different assumptions about the underlying nature of horizontal pleiotropy: MR–Egger regression (intercept and slope terms) [43] and weighted median estimation [44], both of which were employed when there were, at minimum, 3 SNPs in an instrument, and weighted mode estimation [45], which was employed when there were, at minimum, 5 SNPs in an instrument. Additionally, leave-one-out permutation analyses were performed to examine whether any results were driven by individual influential SNPs in IVW models. Lastly, Steiger filtering was employed to orient the direction of causal relationships between presumed risk factors and outcomes for some analyses [46]. This method compares the proportion of risk factor and outcome variance explained by SNPs used as instruments, to help establish whether SNPs associated with both risk factors and outcomes primarily represent (1) a direct association of a SNP with a risk factor, which then influences an outcome, or (2) a direct association of a SNP with an outcome, which then influences the level of a risk factor. Extended descriptions of these sensitivity analyses, along with their assumptions, are provided in S2 Text.

There was no formal prespecified protocol for this study. All analyses described above were decided a priori. All statistical analyses were performed using R version 3.3.1.

This study is reported as per the Strengthening the Reporting of Observational Studies in Epidemiology (STROBE) guideline (S1 STROBE Checklist).

## Results

Across the 12 risk factors that we examined, *F*-statistics for their respective genetic instruments ranged from 24 to 423, suggesting that our analyses were unlikely to suffer from weak instrument bias. For each risk factor, the number of SNPs included in the genetic instrument, along with *R*^2^ and *F*-statistics for the instrument, are provided in Table 1. Complete primary and sensitivity analyses are presented in Table 2 for invasive epithelial ovarian cancer and in S1–S5 Tables for invasive epithelial ovarian cancer histotypes and low malignant potential tumours (subtype-specific analyses). Scatter plots for findings showing strong or suggestive evidence of association in IVW analyses that were consistent in sensitivity analyses are presented in S1 Plots. Leave-one-out plots are presented in S2 Plots.

**Table 2:** IVW and sensitivity analysis estimates for the association of previously reported risk factors with invasive epithelial ovarian cancer risk.

Risk factor	IVW analysis		MR–Egger regression		MR–Egger intercept		Weighted median		Weighted mode	
	OR (95% CI)	*P* value	OR (95% CI)	*P* value	OR (95% CI)	*P* value	OR (95% CI)	*P* value	OR (95% CI)	*P* value
**Reproductive traits**										
Age at menarche	1.07 (1.00–1.14)	0.05	1.00 (0.89–1.13)	0.94	1.00 (1.00–1.01)	0.22	1.01 (0.92–1.10)	0.88	0.98 (0.25–3.84)	0.98
Age at natural menopause	1.03 (1.00–1.06)	0.07	1.07 (1.00–1.14)	0.06	0.99 (0.98–1.00)	0.20	1.03 (0.99–1.07)	0.10	1.04 (1.00–1.09)	0.06
Parity	0.66 (0.26–1.69)	0.39	—	—	—	—	—	—	—	—
**Anthropometric traits**										
Body mass index	1.23 (1.07–1.42)	0.003	1.32 (0.88–1.99)	0.19	1.00 (0.99–1.01)	0.73	1.14 (0.93–1.40)	0.21	1.06 (0.75–1.51)	0.18
Height	1.02 (0.96–1.08)	0.47	1.10 (0.95–1.29)	0.21	1.00 (0.99–1.00)	0.29	1.00 (0.92–1.10)	0.92	0.99 (0.84–1.18)	0.92
**Clinical factors**										
Endometriosis	1.10 (1.06–1.15)	6.9 × 10^−7^	1.25 (1.02–1.53)	0.03	0.99 (0.97–1.01)	0.25	1.06 (1.04–1.09)	6.9 × 10^−6^	1.07 (0.56–2.01)	0.85
Polycystic ovary syndrome	0.99 (0.95–1.04)	0.64	0.93 (0.74–1.16)	0.53	0.99 (0.97–1.01)	0.25	0.97 (0.93–1.02)	0.19	0.97 (0.91–1.04)	0.37
Type 2 diabetes	1.00 (0.97–1.02)	0.81	0.94 (0.81–1.10)	0.46	1.01 (0.99–1.03)	0.48	1.01 (0.97–1.04)	0.72	1.02 (0.96–1.08)	0.52
**Lifestyle factors**										
Lifetime smoking exposure	1.36 (1.04–1.78)	0.02	1.83 (0.65–5.17)	0.26	1.00 (0.99–1.01)	0.57	1.38 (0.93–2.06)	0.11	1.40 (0.55–3.59)	0.48
**Molecular risk factors**										
C-reactive protein	0.97 (0.93–1.02)	0.19	0.99 (0.93–1.06)	0.86	0.99 (0.98–1.01)	0.37	0.98 (0.93–1.03)	0.42	0.98 (0.93–1.03)	0.41
Sex hormone binding globulin	1.09 (0.88–1.35)	0.45	1.22 (0.79–1.89)	0.40	0.99 (0.97–1.01)	0.56	1.14 (0.88–1.48)	0.33	1.14 (0.87–1.49)	0.37
25-hydroxyvitamin D	1.02 (0.72–1.44)	0.93	1.48 (0.82–2.69)	0.28	0.98 (0.95–1.01)	0.24	1.15 (0.85–1.57)	0.36	1.16 (0.84–1.61)	0.41

Estimates are scaled to represent the association of a 1-year decrease in age at menarche; a 1-year increase in age at natural menopause; a 1-child increase in number of children ever born; a 1-SD increase in body mass index (kg/m^2^); a 1-SD increase in height (cm); a 50% higher odds liability to type 2 diabetes, endometriosis, or polycystic ovary syndrome; a 1-unit increase in lifetime smoking exposure; a 1-unit increase in natural-log-transformed 25-hydroxyvitamin D (ng/ml); a 1-unit increase in natural-log-transformed C-reactive protein (mg/l); and a 1-unit increase in natural-log-transformed sex hormone binding globulin (nmol/l). IVW, inverse-variance-weighted; OR, odds ratio.

### Reproductive factors

**Fig 2:**
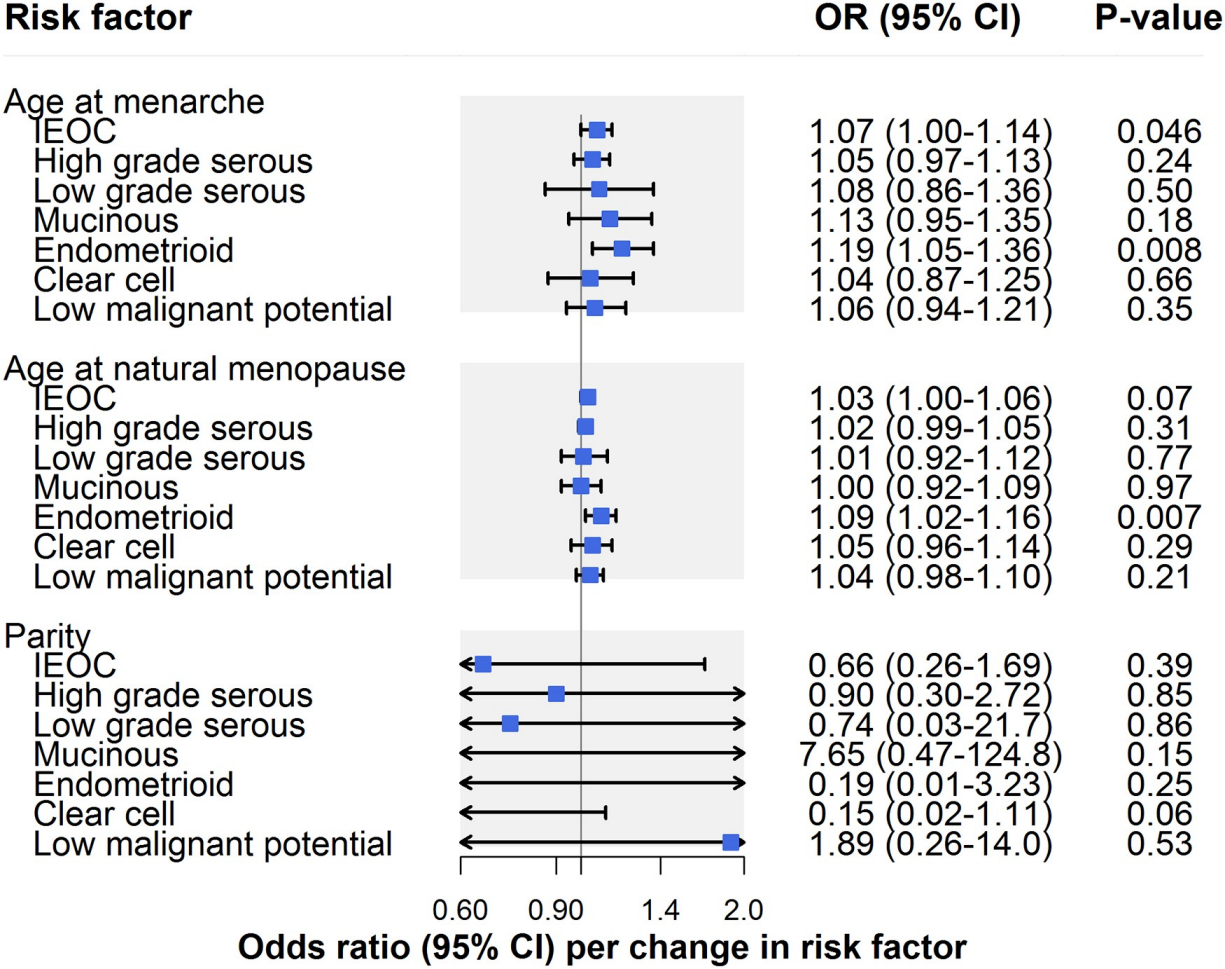
Inverse-variance-weighted estimates for the association of reproductive factors with risk of invasive epithelial ovarian cancer, invasive epithelial ovarian cancer histotypes, and low malignant potential tumours. IEOC, invasive epithelial ovarian cancer; OR, odds ratio. Causal estimates are scaled to represent the effect of a 1-year decrease in age at menarche, a 1-year increase in age at natural menopause, and a 1-child increase in number of children ever born.

In IVW models, there was suggestive evidence for an association of earlier age at menarche with risk of invasive epithelial ovarian cancer (OR per year earlier onset: 1.07, 95% CI 1.00–1.14; *P* = 0.046) (Fig 2; Table 2). However, there was some evidence that horizontal pleiotropy was biasing the IVW estimate. This is because the effect estimate attenuated toward the null when employing MR–Egger regression (OR 1.00, 95% CI 0.89–1.13) and a weighted median estimator (OR 1.01, 95% CI 0.92–1.10) and moved in a protective direction when using a weighted mode estimator (OR 0.98, 95% CI 0.25–3.84). In analyses examining invasive epithelial ovarian cancer histotypes and low malignant potential tumours, there was suggestive evidence for an association of earlier age at menarche with endometrioid carcinoma (OR 1.19, 95% CI 1.05–1.36; *P* = 0.008), which was robust to MR–Egger, weighted median, weighted mode, and leave-one-out analyses (S1 Table).

There was little evidence for an association of later age at natural menopause with risk of invasive epithelial ovarian cancer risk (OR per year later onset: 1.03, 95% CI 1.00–1.06; *P* = 0.07) (Fig 2; Table 2). However, in subtype-specific analyses, there was suggestive evidence for an association of later age at natural menopause with risk of endometrioid carcinoma (OR 1.09, 95% CI 1.02–1.16; *P* = 0.007), which was consistent in sensitivity analyses examining horizontal pleiotropy. While there was little evidence of an association of age at natural menopause with clear cell carcinoma in IVW models (OR 1.05, 95% CI 0.96–1.14; *P* = 0.29), the association strengthened when employing MR–Egger (OR 1.26, 95% CI 1.05–1.52), weighted median (OR 1.11, 95% CI 0.99–1.25), and weighted mode estimators (OR 1.16, 95% CI 1.02–1.31), suggesting horizontal pleiotropy in the IVW model (S1 Table).

In parity analyses, effect estimates were in a protective direction for 5 of 7 ovarian cancer outcomes but were imprecisely estimated, with 95% confidence intervals crossing the null line (Table 2; S1 Table).

### Anthropometric traits

**Fig 3:**
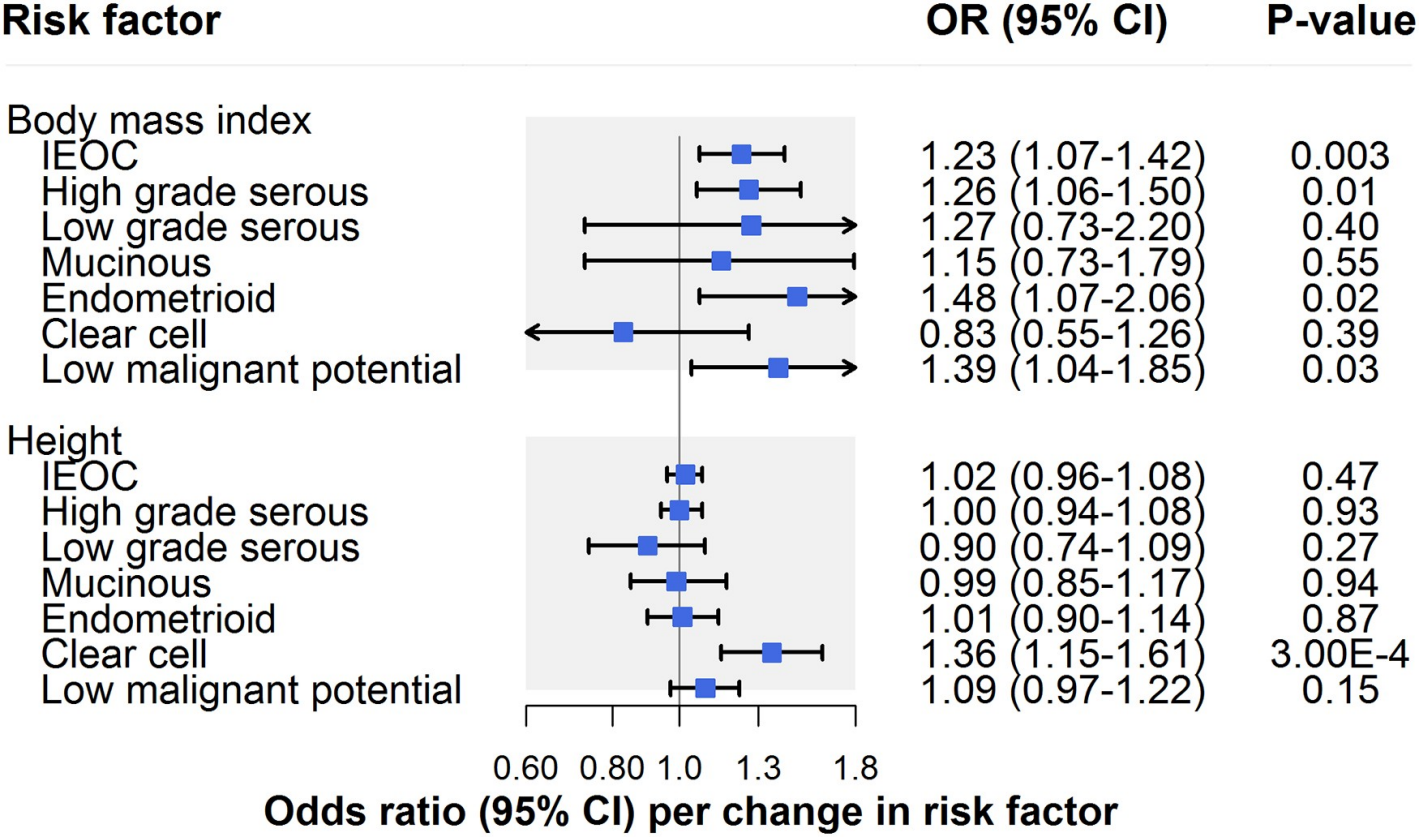
Inverse-variance-weighted estimates for the association of anthropometric traits with risk of invasive epithelial ovarian cancer, invasive epithelial ovarian cancer histotypes, and low malignant potential tumours. IEOC, invasive epithelial ovarian cancer; OR, odds ratio. Causal estimates are scaled to represent the effect of a 1-SD increase in body mass index (kg/m^2^) and a 1-SD increase in height (cm).

There was strong evidence for an association of BMI with invasive epithelial ovarian cancer (OR per 1-SD [4.6 kg/m^2^] increase: 1.23, 95% CI 1.07–1.42; *P* = 0.003) (Fig 3; Table 2). Though there was little evidence for horizontal pleiotropy when performing MR–Egger (OR 1.32, 95% CI 0.88–1.99), inconsistency of effect estimates across weighted median (OR 1.14, 95% CI 0.93–1.40) and weighted mode (OR 1.05, 95% CI 0.75–1.51) approaches suggested potential violations of instrumental variable assumptions.

In IVW models, there was suggestive evidence for an association of BMI with HGSC (OR 1.26, 95% CI 1.06–1.50; *P* = 0.01), endometrioid carcinoma (OR 1.48, 95% CI 1.07–2.06; *P* = 0.02), and low malignant potential tumours (OR 1.39, 95% CI 1.04–1.85; *P* = 0.03) but not with other subtypes. However, there was evidence that horizontal pleiotropy was likely biasing the IVW estimate for HGSC: The effect estimate was attenuated when performing MR–Egger regression (OR 1.05, 95% CI 0.63–1.75) and was inconsistent when employing weighted median (OR 1.17, 95% CI 0.91–1.50) and weighted mode (OR 0.95, 95% CI 0.53–1.35) estimators. Likewise, there was some inconsistency of effect estimates across sensitivity analyses for low malignant potential tumours, with a modest attenuation of the effect estimate observed when employing a weighted mode estimator (OR 1.17, 95% CI 0.55–2.49). Inconsistencies across sensitivity analyses for low malignant potential tumours could reflect horizontal pleiotropy or could reflect limited statistical power for analyses of these tumours. In contrast to HGSC and low malignant potential tumours, the association of BMI with endometrioid carcinoma was also seen across sensitivity analyses using MR–Egger, weighted median, and weighted mode estimators, and in leave-one-out analyses (S2 Table).

There was little evidence for an association of height with invasive epithelial ovarian cancer risk (OR per 1-SD [6.3 cm] increase: 1.02, 95% CI 0.96–1.08; *P* = 0.47) (Fig 3; Table 2). In analyses examining histotypes and low malignant potential tumours, there was strong evidence for an association of height with clear cell carcinoma (OR 1.36, 95% CI 1.15–1.61; *P* = 0.0003), but not with other subtypes. This finding was robust to various sensitivity analyses (S2 Table).

### Clinical factors

**Fig 4:**
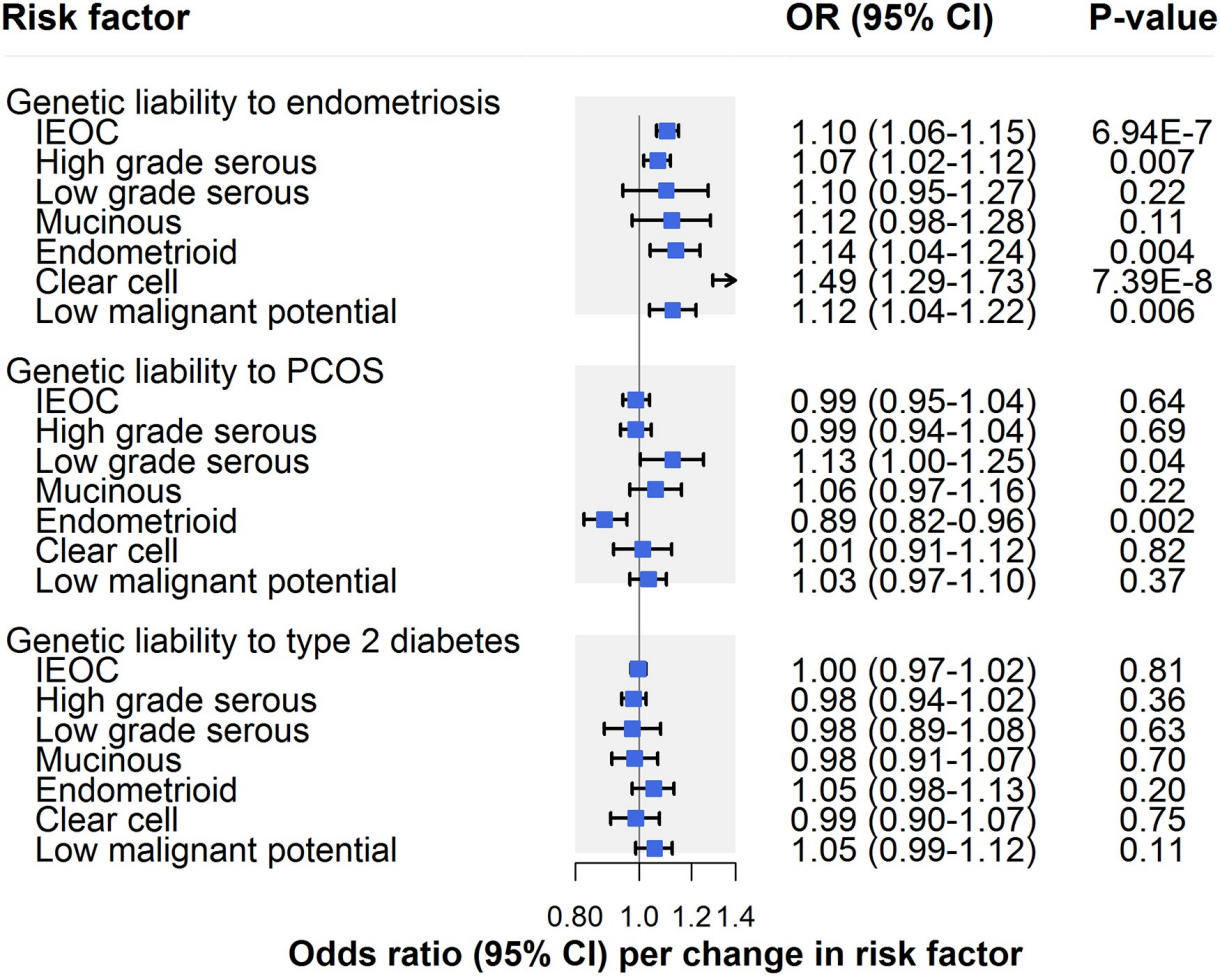
Inverse-variance-weighted estimates for the association of clinical factors with risk of invasive epithelial ovarian cancer, invasive epithelial ovarian cancer histotypes, and low malignant potential tumours. IEOC, invasive epithelial ovarian cancer; OR, odds ratio; PCOS, polycystic ovary syndrome. Causal estimates are scaled to represent the effect of a 50% higher odds liability to endometriosis, PCOS, or type 2 diabetes.

There was strong evidence for an association of genetic liability to endometriosis with invasive epithelial ovarian cancer (OR per 50% higher odds liability to endometriosis: 1.10, 95% CI 1.06–1.15; *P* = 6.94 × 10^−7^), which was consistent across sensitivity analyses examining horizontal pleiotropy (Fig 4; Table 2). In subtype-specific analyses, there was also strong evidence for an association with clear cell carcinoma (OR 1.49, 95% CI 1.29–1.73; *P* = 7.39 × 10^−8^) and suggestive evidence for an association with endometrioid carcinoma (OR 1.14, 95% CI 1.04–1.24; *P* = 0.004), low malignant potential tumours (OR 1.12, 95% CI 1.04–1.22; *P* = 0.006), and HGSC (OR 1.07, 95% CI 1.02–1.12; *P* = 0.007). Findings for clear cell carcinoma were also seen in sensitivity analyses examining horizontal pleiotropy, whereas somewhat inconsistent effect estimates were found for endometrioid carcinoma, low malignant potential tumours, and HGSC. While these inconsistencies could reflect potential violations of instrumental variable assumptions, divergent effect estimates in endometrioid and low malignant potential tumour analyses could also reflect limited statistical power in these analyses (S3 Table). Analyses employing Steiger filtering provided strong evidence that the causal direction between genetic liability to endometriosis and invasive epithelial ovarian cancer was from the former to the latter (*P* = 10^−10^), whereas the causal direction could not be clearly established for clear cell carcinoma analyses (*P* = 0.10).

There was little evidence that genetic liability to PCOS influenced invasive epithelial ovarian cancer risk (OR per 50% higher odds liability to PCOS: 0.99, 95% CI 0.95–1.04; *P* = 0.64) (Fig 4; Table 2). In subtype-specific analyses, there was strong evidence for an inverse association of genetic liability to PCOS with endometrioid carcinoma (OR 0.89, 95% CI 0.82–0.96; *P* = 0.002), which was robust to sensitivity analyses. In contrast, suggestive evidence for an association of PCOS with low grade serous carcinoma (OR 1.13, 95% CI 1.00–1.25; *P* = 0.04) in IVW models was not seen across all sensitivity analyses, suggesting presence of horizontal pleiotropy or potentially reflecting limited statistical power in these analyses. There was little evidence of an association of genetic liability to type 2 diabetes with invasive epithelial ovarian cancer (OR per 50% higher odds liability to type 2 diabetes: 1.00, 95% CI 0.97–1.02; *P* = 0.81) or subtype-specific ovarian cancer (S3 Table).

### Lifestyle factors

**Fig 5:**
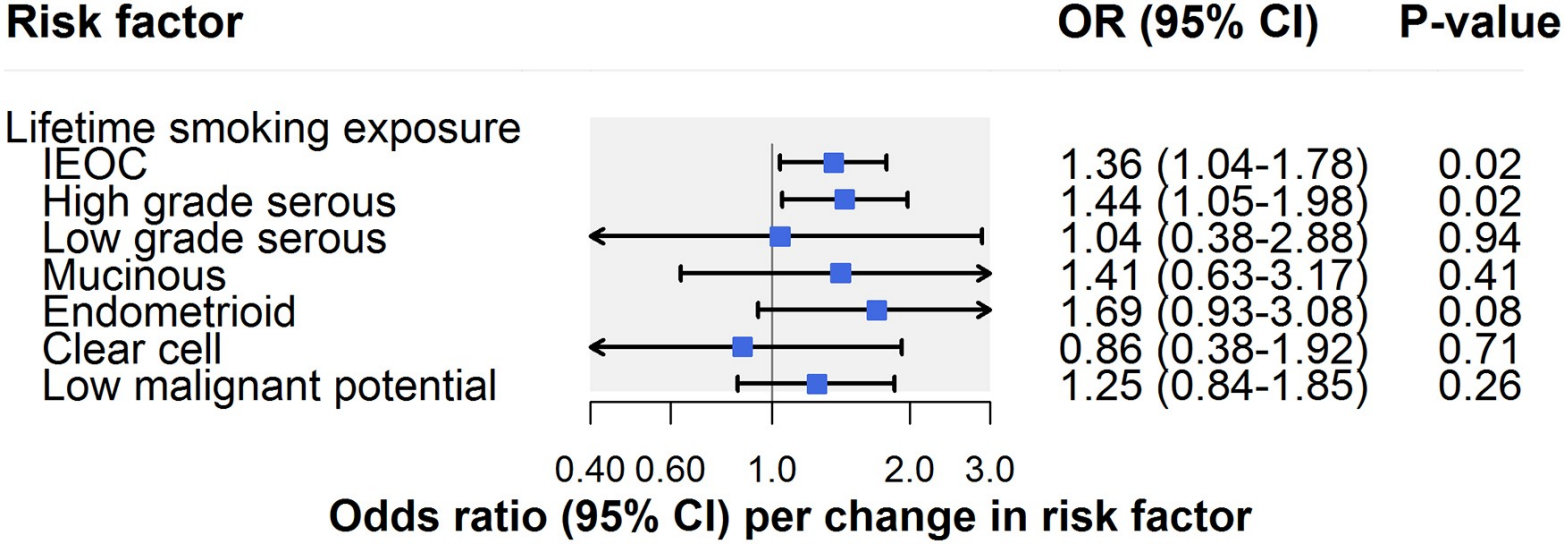
Inverse-variance-weighted estimates for the association of lifestyle factors with risk of invasive epithelial ovarian cancer, invasive epithelial ovarian cancer histotypes, and low malignant potential tumours. IEOC, invasive epithelial ovarian cancer; OR, odds ratio. Causal estimates are scaled to represent the effect of a 1-unit increase in lifetime smoking exposure.

There was suggestive evidence for an association of lifetime smoking exposure with invasive epithelial ovarian cancer (OR per unit increase in smoking score: 1.36, 95% CI 1.04–1.78; *P* = 0.02) (Fig 5; Table 2). A unit increase in smoking score is approximately equivalent to an individual smoking 10 cigarettes per day for 39 years and stopping 16 years ago or an individual smoking 30 cigarettes per day for 14 years and stopping 9 years ago. In subtype-specific analyses, there was also suggestive evidence for an association of smoking with HGSC (OR 1.44, 95% CI 1.05–1.98; *P* = 0.02) but little association with other subtypes. The smoking findings for invasive epithelial ovarian cancer and HGSC were robust to horizontal pleiotropy sensitivity analyses (Tables 2 and S4).

### Molecular risk factors

**Fig 6:**
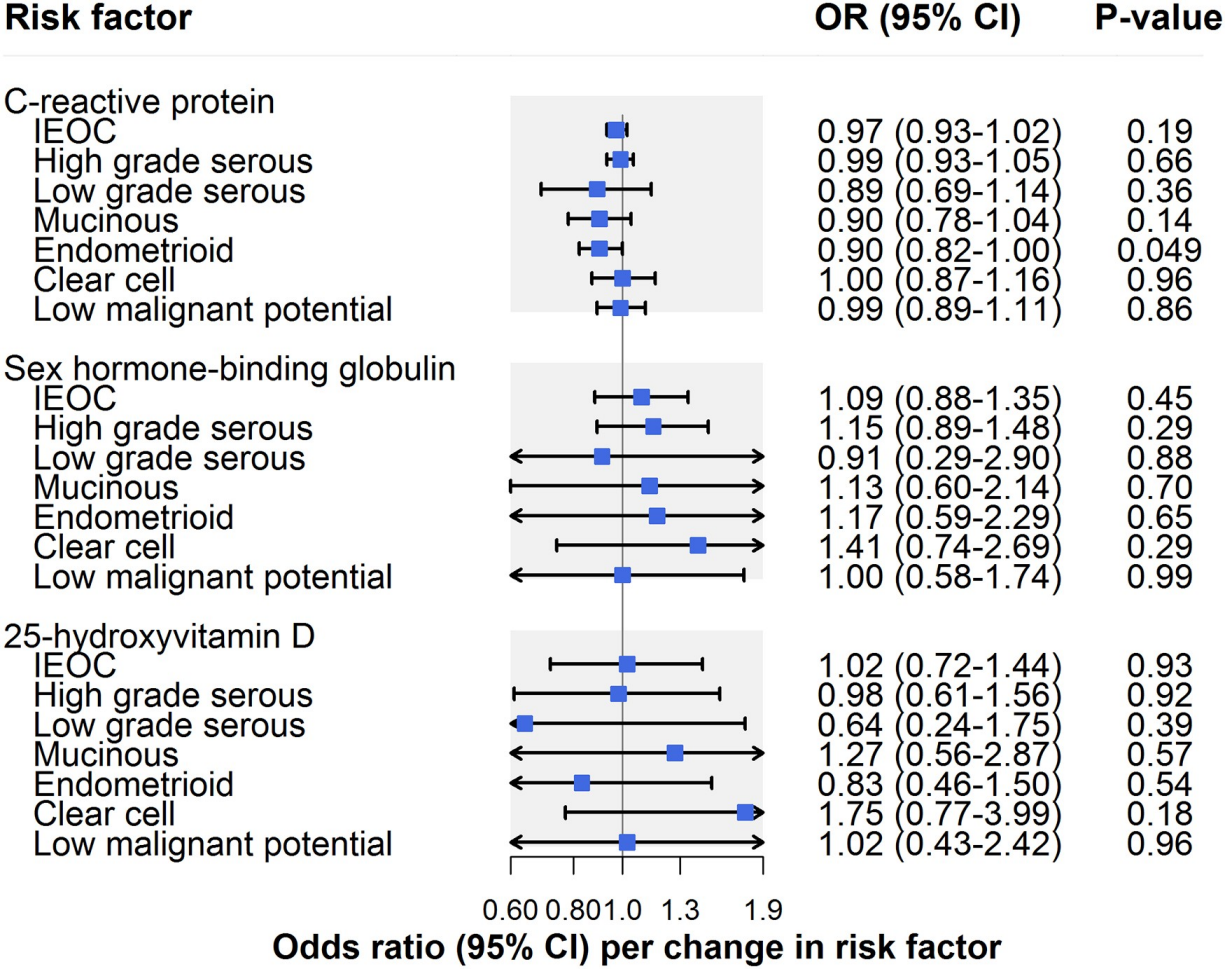
Inverse-variance-weighted estimates for the association of molecular risk factors with risk of invasive epithelial ovarian cancer, invasive epithelial ovarian cancer histotypes, and low malignant potential tumours. IEOC, invasive epithelial ovarian cancer; OR, odds ratio. Causal estimates are scaled to represent the effect of a 1-unit increase in natural-log-transformed C-reactive protein (mg/l), a 1-unit increase in natural-log-transformed sex hormone binding globulin (nmol/l), and a 1-unit increase in natural-log-transformed 25-hydroxyvitamin D (ng/ml).

There was little evidence that CRP influenced invasive epithelial ovarian cancer risk (OR per unit increase in natural log CRP (mg/l): 0.97, 95% CI 0.93–1.02; *P* = 0.19) (Fig 6; Table 2). In analyses examining histotypes and low malignant potential tumours, there was suggestive evidence for an inverse association of CRP with endometrioid carcinoma (OR 0.90, 95% CI 0.82–1.00; *P* = 0.049) (S5 Table). This association was robust to sensitivity analyses using MR–Egger, weighted median, and weighted mode methods in addition to using a restricted CRP instrument (exclusively using 4 SNPs in *CRP*: OR 0.72, 95% CI 0.42–1.22; *P* = 0.14). CRP was not clearly associated with other histotypes or with low malignant potential tumours. There was no strong or suggestive evidence for an association of sex hormone binding globulin (OR per unit increase in natural-log-transformed sex hormone binding globulin [nmol/l]: 1.09, 95% CI 0.88–1.35; *P* = 0.45) or circulating 25-hydroxyvitamin D (OR per unit increase in natural-log-transformed 25-hydroxyvitamin D [ng/ml]: OR 1.02, 95% CI 0.72–1.44; *P* = 0.93) with invasive epithelial ovarian cancer risk (Fig 6; Table 2) or with subtype-specific ovarian cancer (S5 Table).

### Discussion

This MR analysis of up to 66,450 women supports associations of genetic liability to endometriosis and lifetime smoking exposure with invasive epithelial ovarian cancer risk but found little clear evidence to support roles of 10 other previously reported risk factors in ovarian carcinogenesis. In analyses examining invasive epithelial ovarian cancer histotypes and low malignant potential tumours, there was strong or suggestive evidence for associations of ages at menarche and natural menopause, BMI, height, lifetime smoking exposure, CRP, and genetic liabilities to endometriosis and PCOS with ovarian cancer risk. There was little evidence to support associations of genetic liability to type 2 diabetes, parity, or circulating levels of 25-hydroxyvitamin D or sex hormone binding globulin with the various ovarian cancer outcomes assessed.

Though historically considered a homogeneous disease with a single cellular origin, epithelial ovarian cancer is now recognised as heterogeneous, consisting of multiple histological subtypes, each with its own distinct origins, morphological characteristics, and molecular alterations [22,47–50]. The largely histotype-specific findings in this analysis using genetic variants as proxies to minimise confounding and avoid reverse causation bias thus help to extend these insights further by supporting distinct causal pathways across invasive epithelial ovarian cancer histotypes.

Some of the histotype-specific findings are consistent with previous studies. For example, in agreement with previous observational and MR analyses [7–10,13,51,52], most risk factors did not show clear evidence of association with HGSC. Consistent with some studies, age at natural menopause was most strongly associated with endometrioid carcinoma [8], and height was most strongly associated with clear cell carcinoma [14,53]. While our finding of little evidence of an association of BMI with HGSC is consistent with previous observational and MR analyses, we were unable to examine—using the summarised genetic association data available to us—whether the previously reported 29% (95% CI 3%–61%) increased odds of non-HGSC (per 5-unit increase in genetically proxied BMI) was replicated in our sample [13,54].

Some findings in this MR analysis were not consistent with those observed in previous conventional observational and MR analyses. Most notably, previously reported associations between smoking and mucinous carcinoma [9,55–57] were not corroborated in MR analyses of lifetime smoking exposure. Though estimates from primary and sensitivity analyses all included the null line, inconsistencies in effect estimates across these analyses support pleiotropic biases distorting the causal effect estimate, though we cannot rule out that divergent effect estimates could also reflect the unit change in lifetime smoking exposure that our analyses are scaled to. Hypothetically, analyses scaled to a smaller unit change in lifetime smoking exposure would be expected to show greater convergence of effect estimates around the null, providing less appearance of “inconsistency” across effect estimates. Though parity has been consistently inversely associated with risk of ovarian cancer in conventional analyses [10,58–62], MR effect estimates suggesting a protective association of giving birth to more children were imprecise, and 95% confidence intervals spanned the null line. Given the few SNPs available to proxy for parity (2 independent variants in this analysis), these results likely reflect limited statistical power. Though genetically proxied height has previously been associated with increased risk of both invasive epithelial ovarian cancer (OR per 5-cm increase in height: 1.06, 95% CI 1.01–1.11) and low malignant potential tumours (OR 1.15, 95% CI 1.02–1.29), we found little consistent evidence for associations with height across our primary and sensitivity analyses, though 95% confidence intervals overlapped across both studies [14]. While this previous individual-data MR analysis was smaller than ours (16,395 versus 22,406 cases), the authors constructed genetic instruments from individual-level data for height using 609 SNPs (versus 345 SNPs in our analysis), which may have afforded this previously reported analysis greater instrument strength and, thus, greater statistical power to detect effects reported. We restricted our analysis to 345 independent variants in order to allow us to employ various summary-level data sensitivity analyses that require independent variants in the instrument. Likewise, consistent with a previous MR analysis of 25-hydroxyvitamin D and multiple-site cancer risk (including ovarian cancer) [63], we were unable to replicate previously reported inverse associations of genetically proxied 25-hydroxyvitamin D with epithelial ovarian cancer (OR per 20-nmol/l increase in 25-hydroxyvitamin D: 0.79, 95% CI 0.66–0.94) or HGSC (OR 0.65, 95% CI 0.50–0.84) [15]. When we employed the same 3 variants used to proxy 25-hydroxyvitamin D in this other study, along with the same SNP–exposure estimates within our analysis, we likewise found little evidence of association with invasive epithelial ovarian cancer (OR per 20-nmol/l increase: 0.91, 95% CI 0.81–1.04) or HGSC (OR 0.90, 95% CI 0.77–1.04).

Our MR analyses found some evidence for an unexpected inverse association of CRP, a marker of systemic inflammation, with endometrioid carcinoma. Given recent evidence suggesting a role of infectious agents in ovarian cancer [64,65], a possible protective effect of CRP on endometrioid carcinoma could speculatively reflect the involvement of CRP in acute immune response (i.e., protection against active bacterial and viral infections).

Overall, few previously reported risk factors showed clear evidence of a role in invasive epithelial ovarian cancer or HGSC, the most common (approximately 70% of cases) and lethal histotype, suggesting that some previously reported associations may have been driven by residual confounding, misclassification biases, or reverse causation [66]. A notable exception was suggestive evidence that smoking increased the odds of HGSC, consistent with some [67,68], but not all [9,55,64,65], observational analyses. An association of genetic liability to endometriosis with invasive epithelial ovarian cancer corroborates findings from conventional analyses that women with this condition are at elevated risk of subsequent disease [9,69]. This finding also suggests that subclinical manifestations of or pathways leading to endometriosis may influence oncogenesis, indicating important avenues for future mechanistic work. While observational and MR estimates examining associations between measures of endometriosis and risk of invasive epithelial ovarian cancer are qualitatively similar (i.e., in the same direction), it is important to emphasise that observational effect estimates for disease states examined in this analysis (i.e., endometriosis, PCOS, type 2 diabetes) cannot be compared quantitatively (i.e., in direction and magnitude) to the MR estimates presented in this analysis as the latter are examining the association of genetic liability to disease (i.e., subclinical manifestations of or pathways leading to the disease), rather than presence of disease per se.

It is important to note that while the risk factors included in this analysis have all been identified in systematic or narrative reviews and cancer prevention guidelines as being potentially implicated in ovarian cancer development, there are varying degrees of evidence from the observational literature supporting their role in cancer risk. For example, while both the WCRF/AICR 2014 report and Cancer Research UK (https://www.cancerresearchuk.org/about-cancer/ovarian-cancer/risks-causes; accessed on 4 September 2019) state that excess body fatness is linked to ovarian cancer, only the WCRF/AICR 2014 report finds “convincing evidence” that adult attained height increases risk of this disease. Nulliparity and endometriosis have been consistently associated with elevated ovarian cancer risk in observational studies [8–10,69], whereas there is weaker evidence linking earlier age at menarche to cancer risk [8–10]. Though current cigarette smoking has been strongly linked to mucinous ovarian cancer in large pooled analyses, associations with other subtypes have been less consistent [64,65]. Given that few well-powered studies to date have stratified analyses across common ovarian cancer subtypes, it is a challenge to develop a consensus on the current strength of evidence linking previously reported risk factors to these subtypes.

Strengths of this analysis include the use of a systematic approach to collate previously reported risk factors for epithelial ovarian cancer, the appraisal of the role of these risk factors in disease aetiology using a MR framework to reduce confounding and avoid reverse causation bias, the employment of complementary sensitivity analyses to rigorously assess for violations of MR assumptions, and the restriction of datasets utilised to women of primarily or exclusively European descent to minimise confounding through population stratification.

There are several limitations to these analyses. First, though *F*-statistics generated for most risk factors suggested that results were unlikely to suffer from weak instrument bias, statistical power for some analyses of less common ovarian cancer subtypes (low grade serous, mucinous, and clear cell carcinomas) was likely modest, meaning that the possibility that some results may reflect “false negative” findings cannot be ruled out. This limited statistical power may account for instances where risk factors showed strong or suggestive evidence of association for a single subtype but for which there was little apparent evidence of heterogeneity in effect estimates across all subtypes assessed (e.g., for age at menarche and age at natural menopause analyses). As such, it is important to emphasise that for some risk factors, subtype-specific findings are more likely to reflect differences in statistical power across subtypes than genuine differences in the aetiological role of that risk factor in disease onset. Further, given the low statistical power of sensitivity analyses employed to examine horizontal pleiotropy (MR–Egger, weighted median, and weighted mode estimators), particularly for analyses of less common ovarian cancer subtypes, we were unable to robustly discriminate whether divergent effect estimates across sensitivity analyses in these subtypes reflected evidence of horizontal pleiotropy or limited statistical power. Since analyses were performed using summarised genetic association data in aggregate, it was not possible to restrict age at natural menopause analyses exclusively to participants who had undergone menopause. However, given that most ovarian cancer cases occur after menopause and that age-matched controls were used, the inclusion of some pre- or perimenopausal women in these analyses would likely have biased results toward the null (i.e., providing a conservative effect estimate). A genetic instrument for lifetime smoking exposure was constructed from genome-wide association analyses reported in a preprint [38]. As with all analyses that have not been formally peer-reviewed, findings from preprints may be subject to change prior to publication. Though a multiple-testing correction was applied to findings from analyses to account for the 12 risk factors examined, false positive findings arising from the number of analyses performed across ovarian cancer outcomes cannot be ruled out, especially as statistical power was limited in analyses of histotypes. Additionally, all models employed assumed no interaction (e.g., gene–environment, gene–gene) or effect modification, and linear relationships between risk factors and ovarian cancer. Lastly, the use of a MR framework precluded directly examining the role of some ovarian cancer risk factors that do not have robust genetic variants available to serve as proxies (e.g., use of oral contraceptives, hormone replacement therapy).

Though the largely null findings for invasive epithelial ovarian cancer in this analysis can assist in de-prioritising certain intervention targets for ovarian cancer prevention, they also underscore the challenges in establishing effective primary prevention strategies for this malignancy. To date, beyond risk-reducing surgical interventions, only regular use of oral contraceptive pills has shown compelling evidence of reducing the risk of subsequent disease [61,70,71]. The continued identification of robust genetic variants to proxy other lifestyle and molecular factors previously reported to influence ovarian cancer (e.g., additional sex hormones, gonadotropins, inflammatory markers) will allow for a more refined assessment of the influence of these factors in ovarian carcinogenesis [51,72]. Additionally, further work to understand the possible mechanisms through which factors that appear to influence ovarian cancer in these analyses promote oncogenesis (e.g., genetic liability to endometriosis, low CRP levels) could help to increase the scope for prevention opportunities across the life course. Lastly, for the vast majority of women who develop ovarian cancer with no previous history of smoking and who do not have endometriosis [9,55,73], there is a need to identify novel modifiable risk factors for this condition, as has been advocated elsewhere [74,75].

### Conclusions

Of 12 previously reported risk factors examined for association with invasive epithelial ovarian cancer, only genetic liability to endometriosis and lifetime smoking exposure showed evidence of association with disease risk. When stratified on histotype and low malignant potential tumours, most risk factors were associated with 1 or more subtypes, underscoring the heterogeneous nature of this disease. While this aetiological heterogeneity could have implications for understanding mechanisms of tumour pathology and for studies examining histotype-specific prognosis, given the low incidence of epithelial ovarian cancer in the general population, prevention strategies targeting factors causally implicated in overall epithelial ovarian cancer risk are most likely to confer important population-level reductions in disease incidence. Along with effective clinical management of endometriosis and policies to prevent the initiation of tobacco use and encourage smoking cessation, established prevention strategies like the use of oral contraceptives continue to be an important mechanism for reducing ovarian cancer risk. The identification of novel modifiable risk factors remains an important priority for the control of epithelial ovarian cancer.

## Supporting information

S1 Data Summary genetic association data used to perform analyses. (XLSX)

S1 Plots Scatter plots for findings showing strong or suggestive evidence of association in IVW analyses that were consistent in sensitivity analyses. (PDF)

S2 Plots Leave-one-out permutation analyses. (PDF)

S1 STROBE Checklist STROBE reporting checklist. (DOC)

S1 Table IVW and sensitivity analysis estimates for the association of reproductive factors with risk of invasive epithelial ovarian cancer histotypes and low malignant potential tumours. (DOCX)

S2 Table IVW and sensitivity analysis estimates for the association of anthropometric traits with risk of invasive epithelial ovarian cancer histotypes and low malignant potential tumours. (DOCX)

S3 Table IVW and sensitivity analysis estimates for the association of clinical factors with risk of invasive epithelial ovarian cancer histotypes and low malignant potential tumours. (DOCX)

S4 Table IVW and sensitivity analysis estimates for the association of lifestyle factors with risk of invasive epithelial ovarian cancer histotypes and low malignant potential tumours. (DOCX)

S5 Table IVW and sensitivity analysis estimates for the association of molecular risk factors with risk of invasive epithelial ovarian cancer histotypes and low malignant potential tumours. (DOCX)

S1 Text Description of OCAC and OncoArray analyses, complete PubMed and Web of Science search strategies, review papers identified from PubMed and Web of Science searches, risk factors ascertained for inclusion, and risk factor inclusion stages. (DOCX)

S2 Text Description of GWAS and genetic instrument selection, sensitivity analyses to examine horizontal pleiotropy, assessment of sex-specific instruments, and risk-factor-specific sensitivity analyses. (DOCX)

## Abbreviations

BMI: body mass index
CRP: C-reactive protein
GWAS: genome-wide association study
HGSC: high grade serous carcinoma
IVW: inverse-variance-weighted
MR: Mendelian randomization
OCAC: Ovarian Cancer Association Consortium
OR: odds ratio
PCOS: polycystic ovary syndrome
WCRF/AICR: World Cancer Research Fund/American Institute for Cancer Research
