# Supplementary material for: Appraising the role of previously reported risk factors in epithelial ovarian cancer risk: A Mendelian randomization analysis
Source: PLoS Med. 2019 Aug 7;16(8):e1002893. doi: 10.1371/journal.pmed.1002893 (PMC6685606; doi:10.1371/journal.pmed.1002893)
Supplement: S1 Plots — (PDF) [file pmed.1002893.s002.pdf]

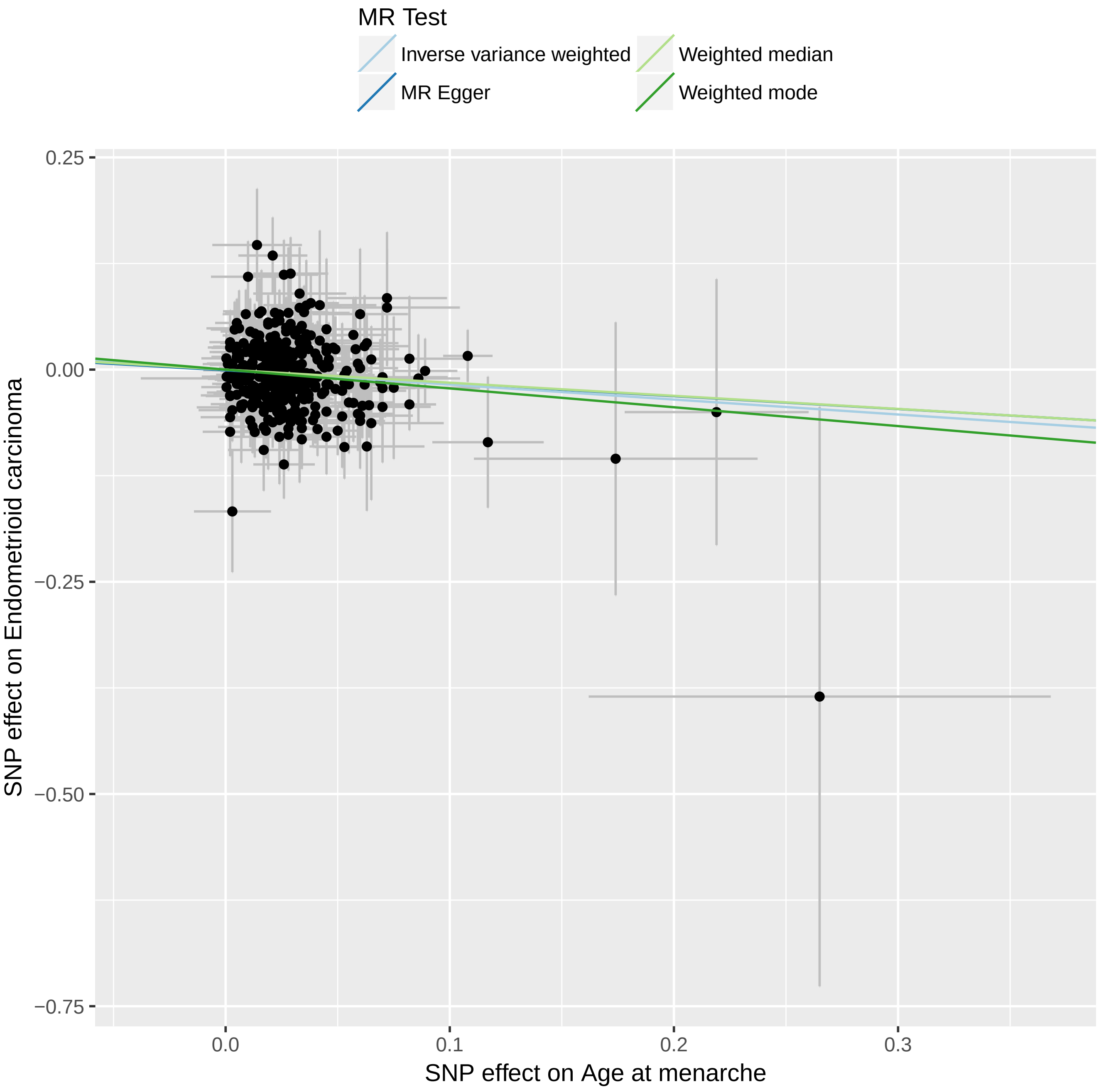
MR Test
Inverse variance weighted
Weighted median
MR Egger
Weighted mode
0.25
0.00
−0.25
−0.50
−0.75
0.0
0.1
0.2
0.3
SNP effect on Endometrioid carcinoma
SNP effect on Age at menarche

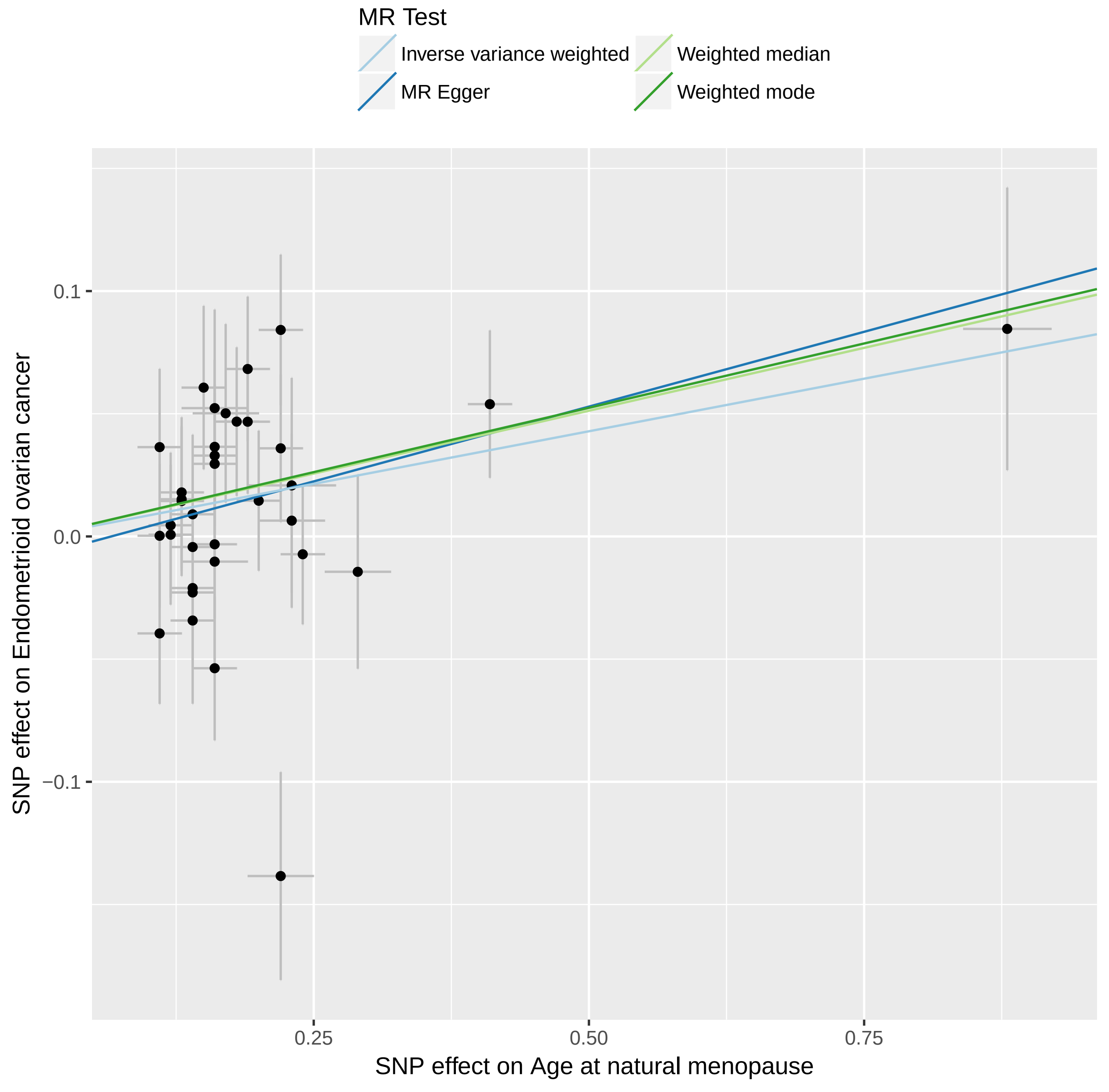

MR Test
Inverse variance weighted
Weighted median
MR Egger
Weighted mode
0.1
0.0
−0.1
0.25
0.50
0.75
SNP effect on Endometrioid ovarian cancer
SNP effect on Age at natural menopause

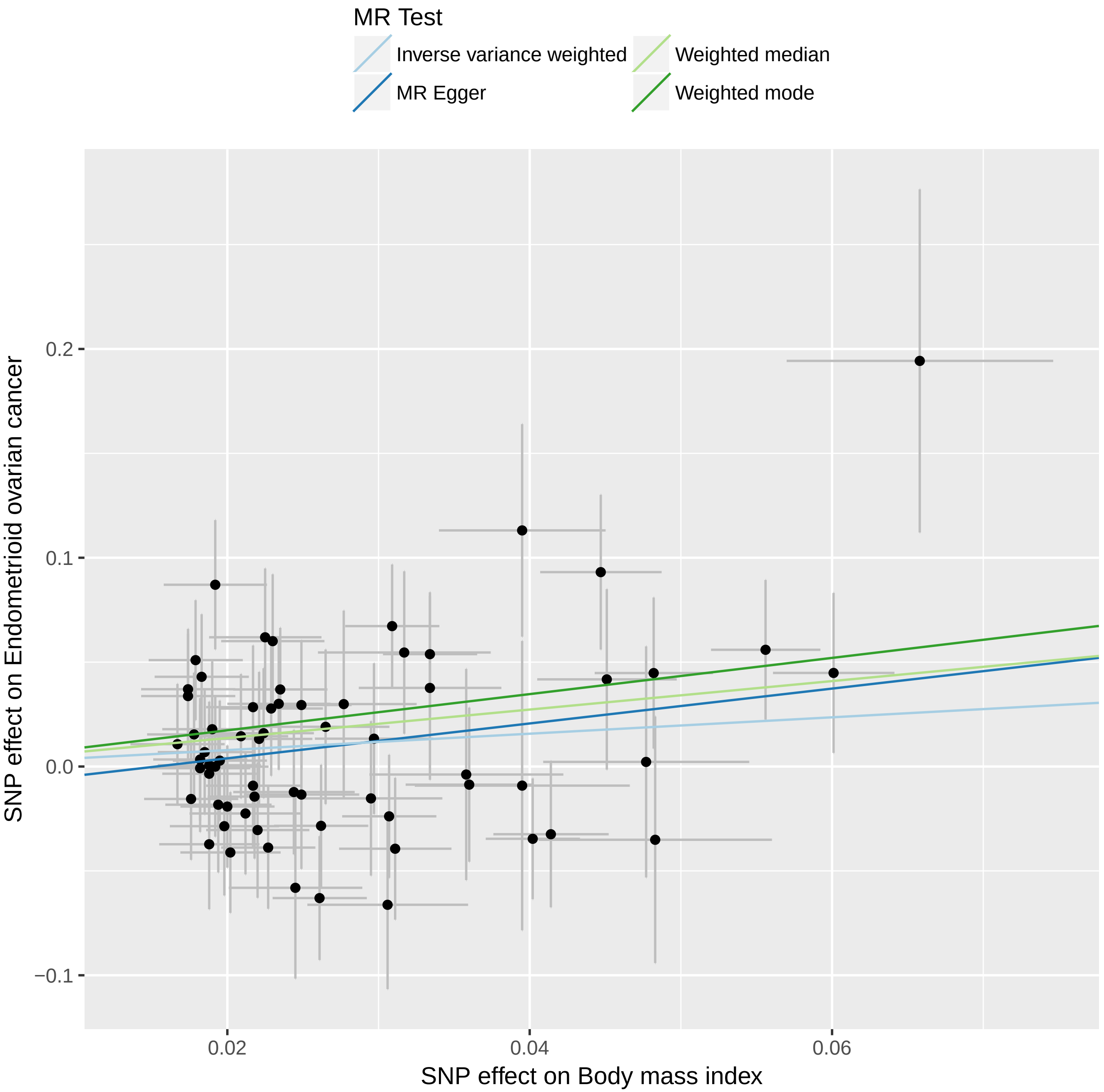
MR Test
Inverse variance weighted
MR Egger
Weighted median
Weighted mode
0.2
0.1
0.0
−0.1
0.02
0.04
0.06
SNP effect on Endometrioid ovarian cancer
SNP effect on Body mass index

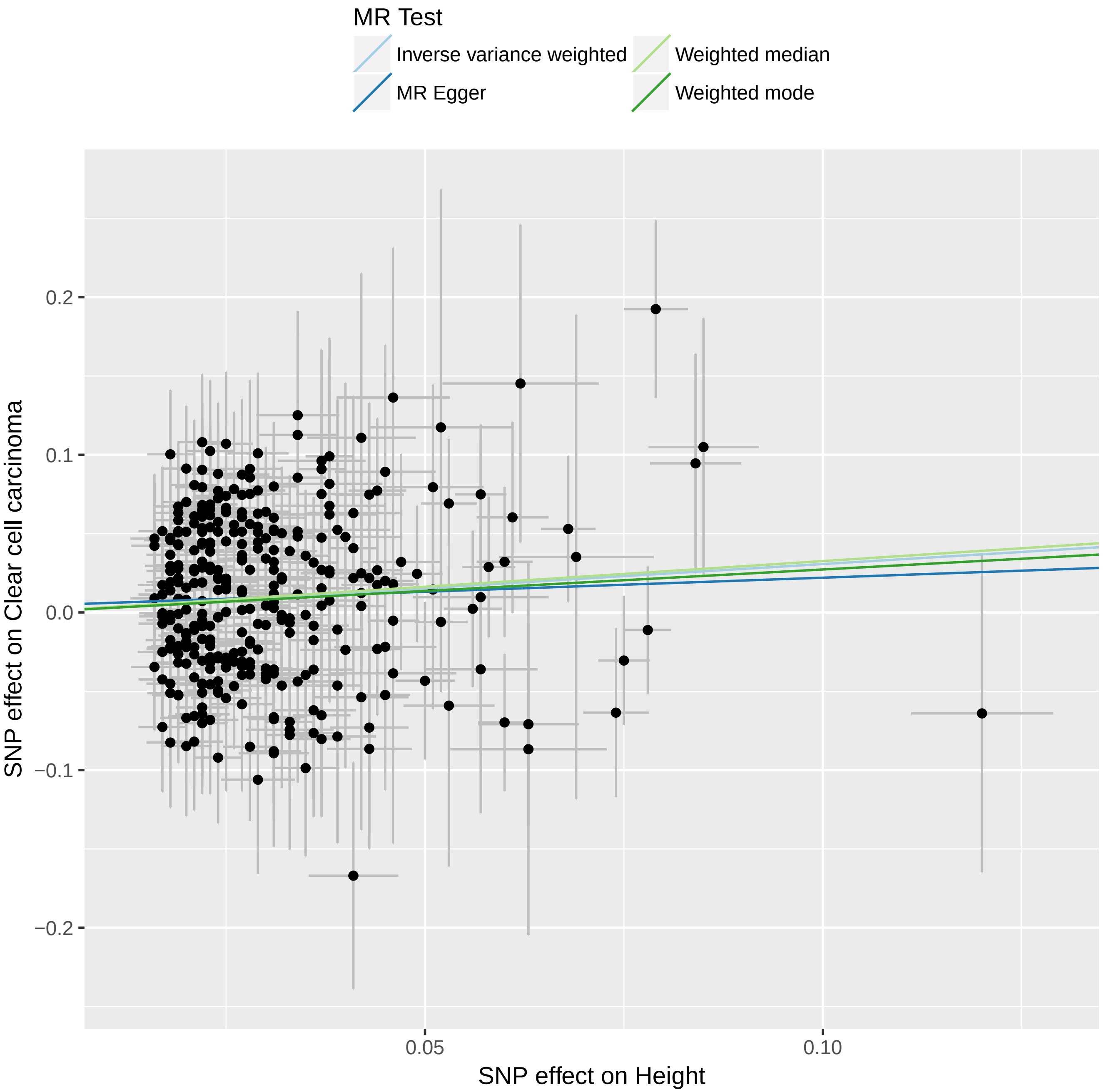

MR Test
Inverse variance weighted
Weighted median
MR Egger
Weighted mode
SNP effect on Clear cell carcinoma
0.2
0.1
0.0
−0.1
−0.2
0.05
0.10
SNP effect on Height

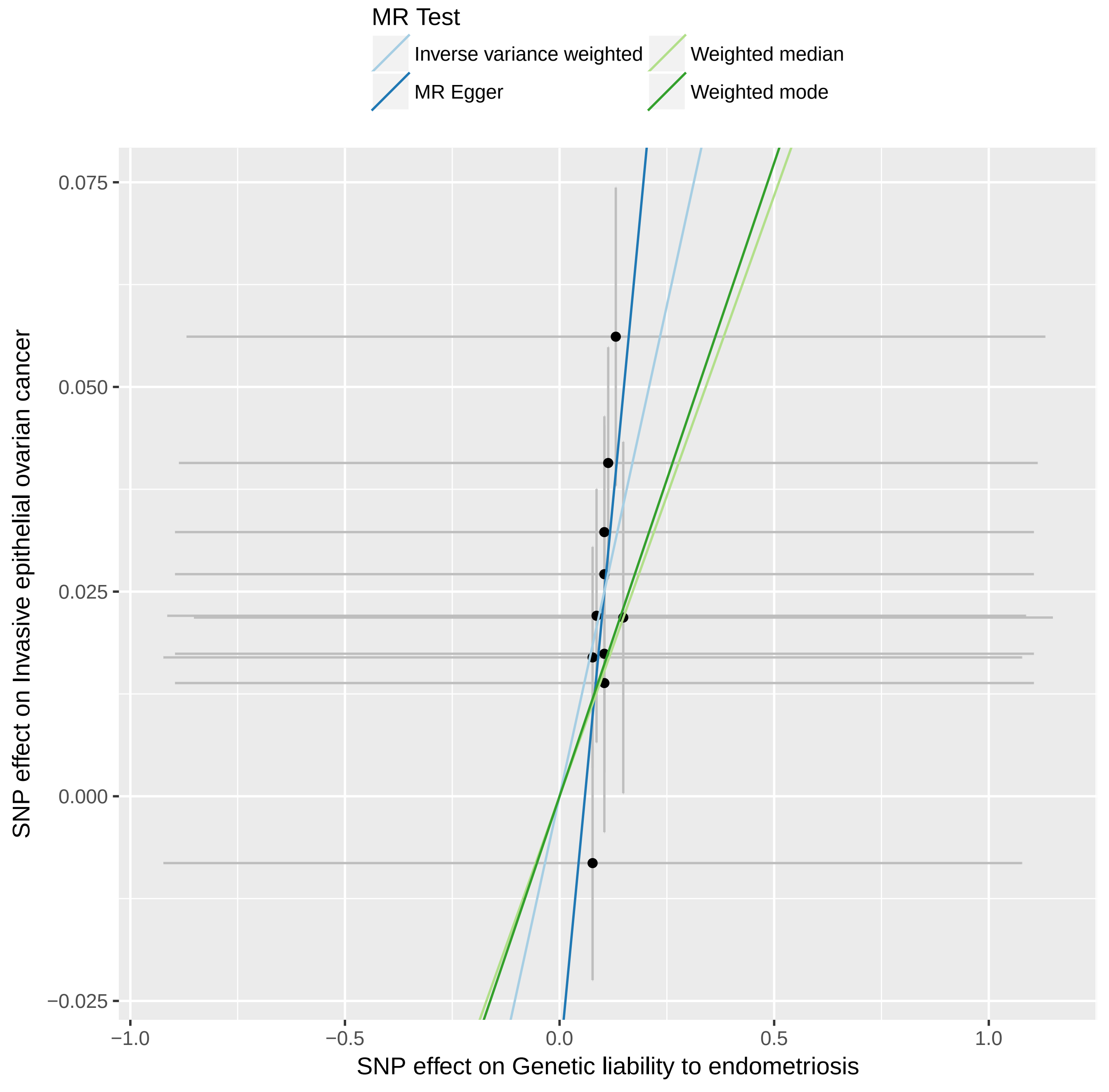

MR Test
Inverse variance weighted
Weighted median
MR Egger
Weighted mode
SNP effect on Invasive epithelial ovarian cancer
0.075
0.050
0.025
0.000
−0.025
−1.0
−0.5
0.0
0.5
1.0
SNP effect on Genetic liability to endometriosis

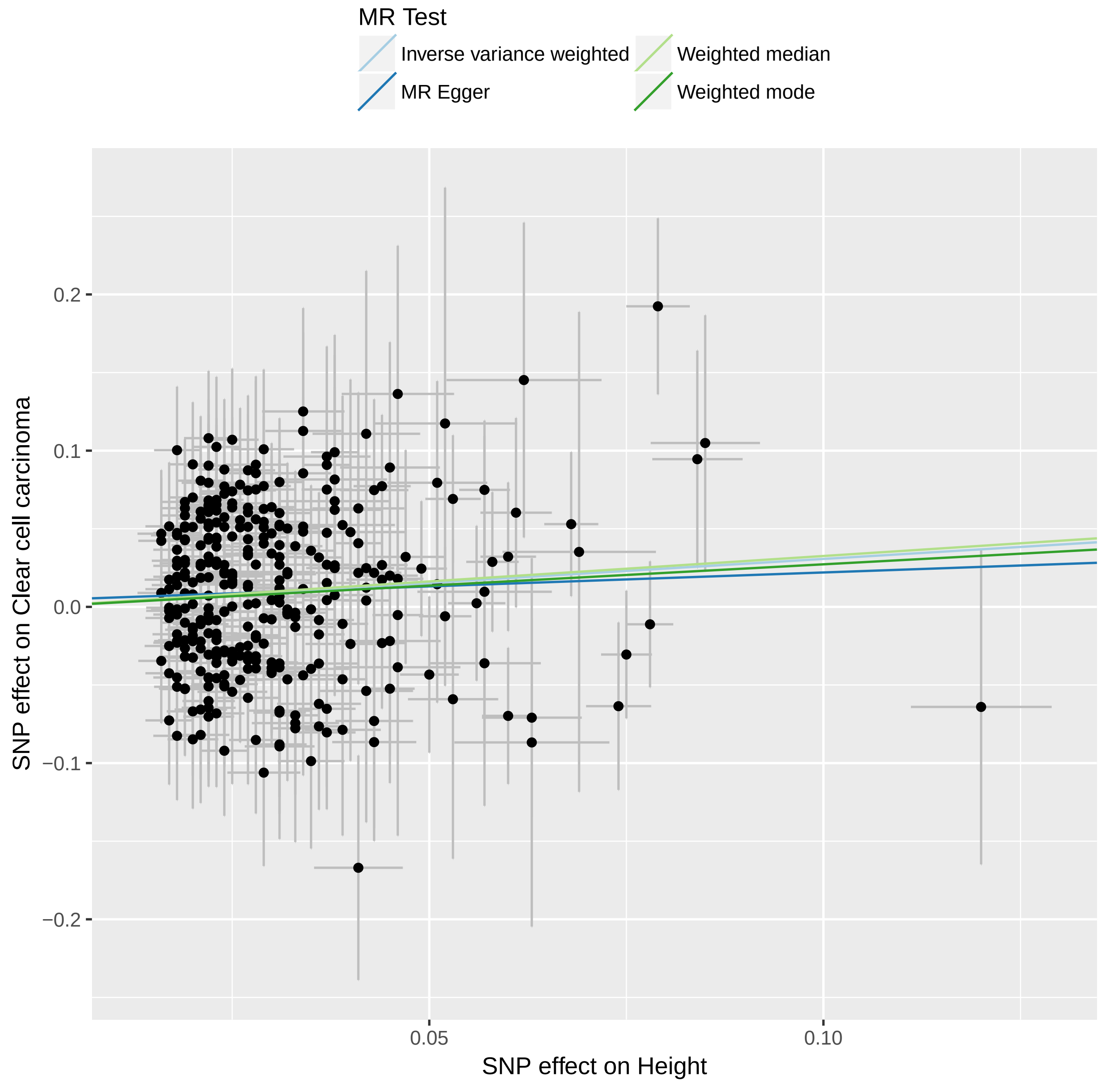

MR Test
Inverse variance weighted
Weighted median
MR Egger
Weighted mode
0.2
0.1
0.0
−0.1
−0.2
0.05
0.10
SNP effect on Clear cell carcinoma
SNP effect on Height

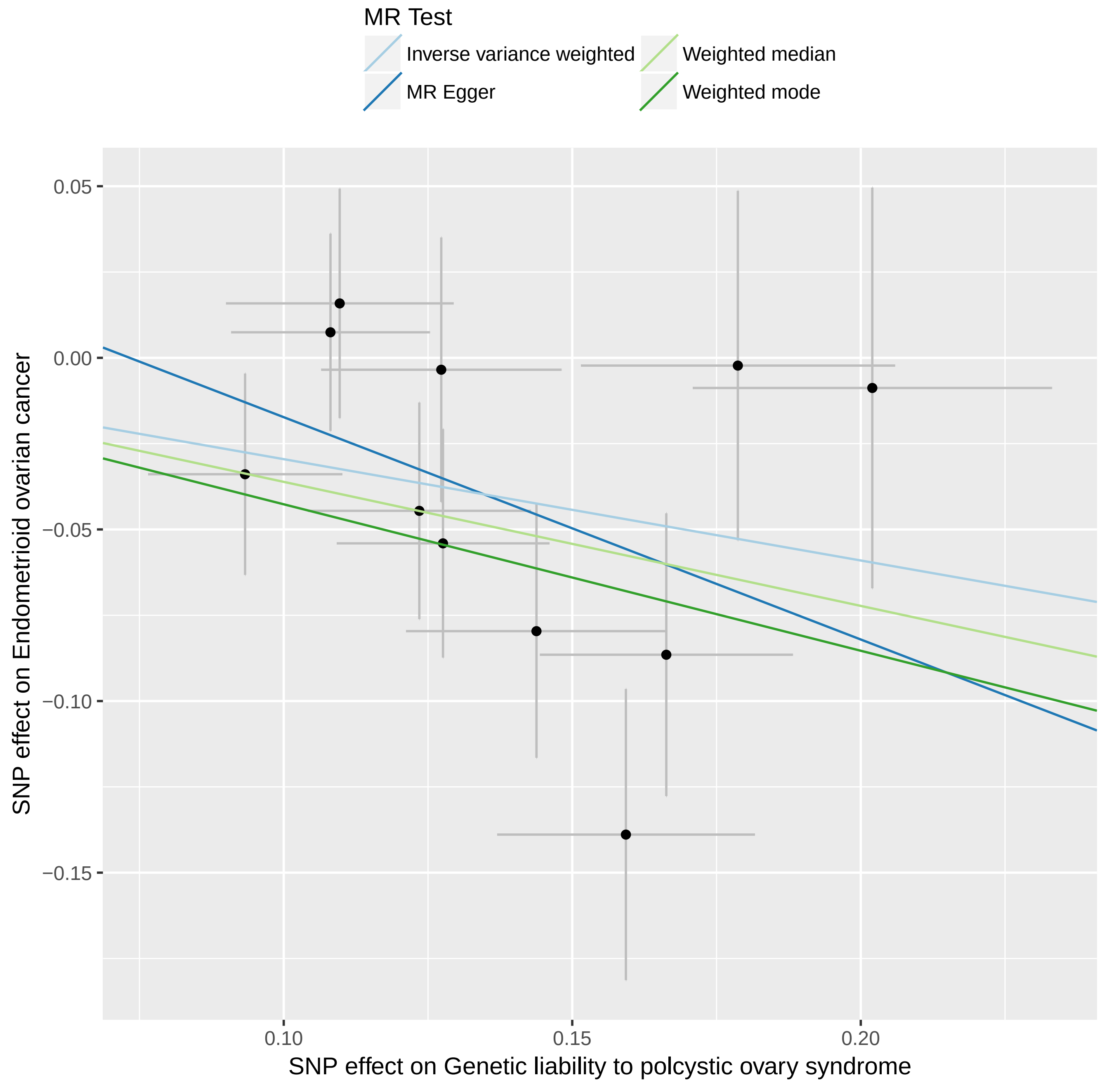
MR Test
Inverse variance weighted
Weighted median
MR Egger
Weighted mode
0.05
0.00
−0.05
−0.10
−0.15
0.10
0.15
0.20
SNP effect on Endometrioid ovarian cancer
SNP effect on Genetic liability to polcystic ovary syndrome

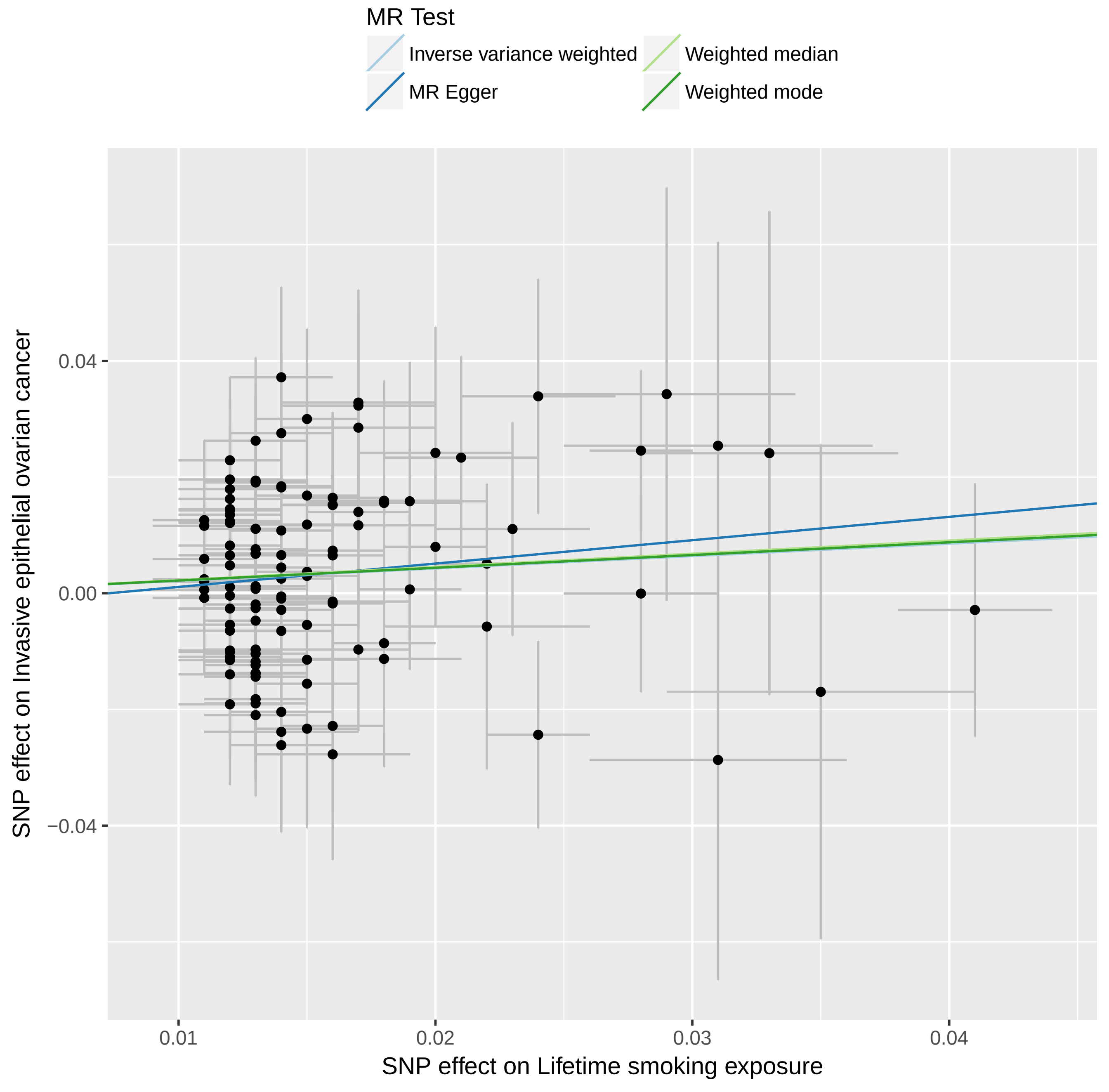
MR Test
Inverse variance weighted
Weighted median
MR Egger
Weighted mode
SNP effect on Invasive epithelial ovarian cancer
0.04
0.00
−0.04
0.01
0.02
0.03
0.04
SNP effect on Lifetime smoking exposure

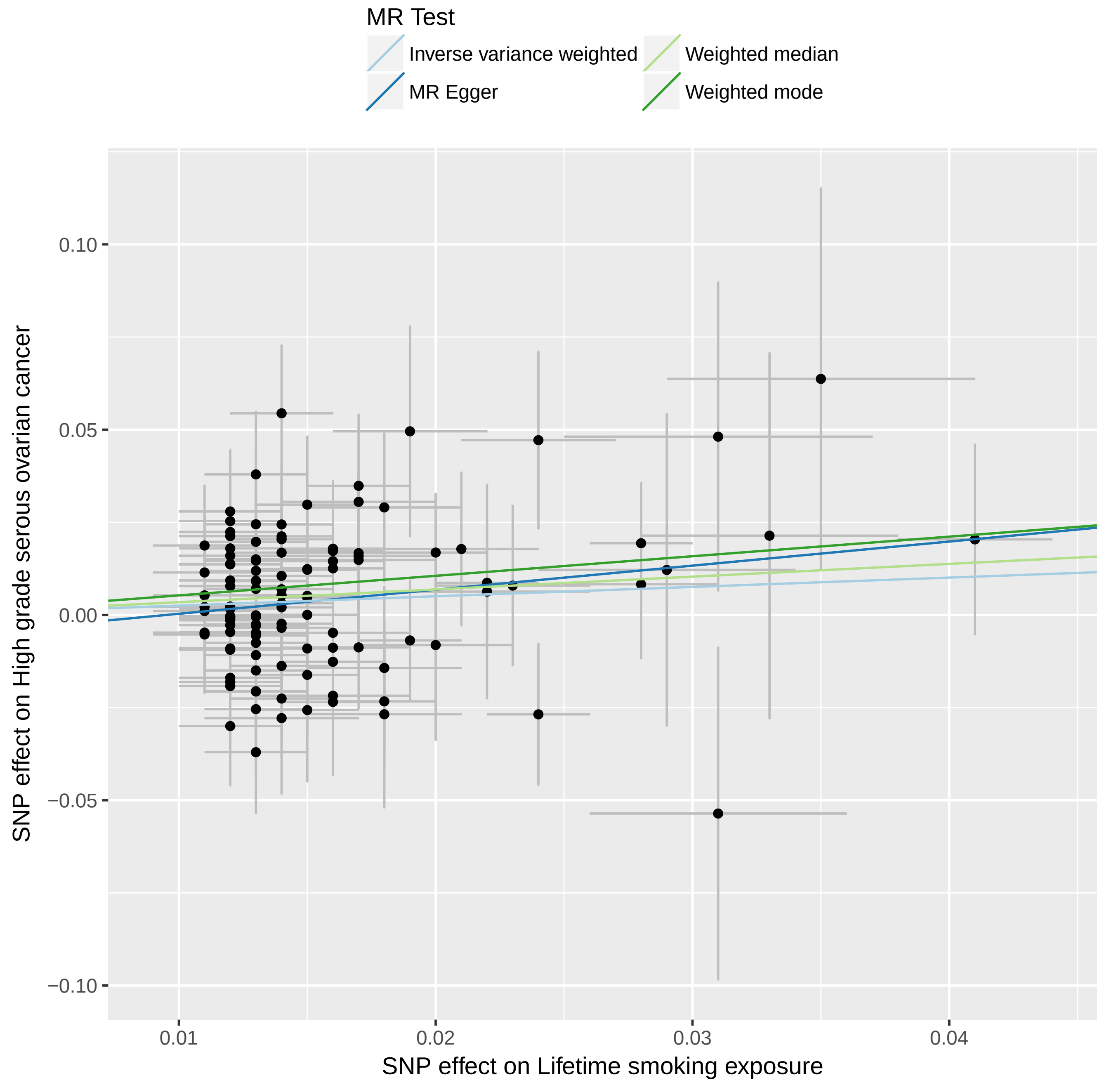

MR Test
Inverse variance weighted
MR Egger
Weighted median
Weighted mode
0.10
0.05
0.00
−0.05
−0.10
SNP effect on High grade serous ovarian cancer
0.01
0.02
0.03
0.04
SNP effect on Lifetime smoking exposure

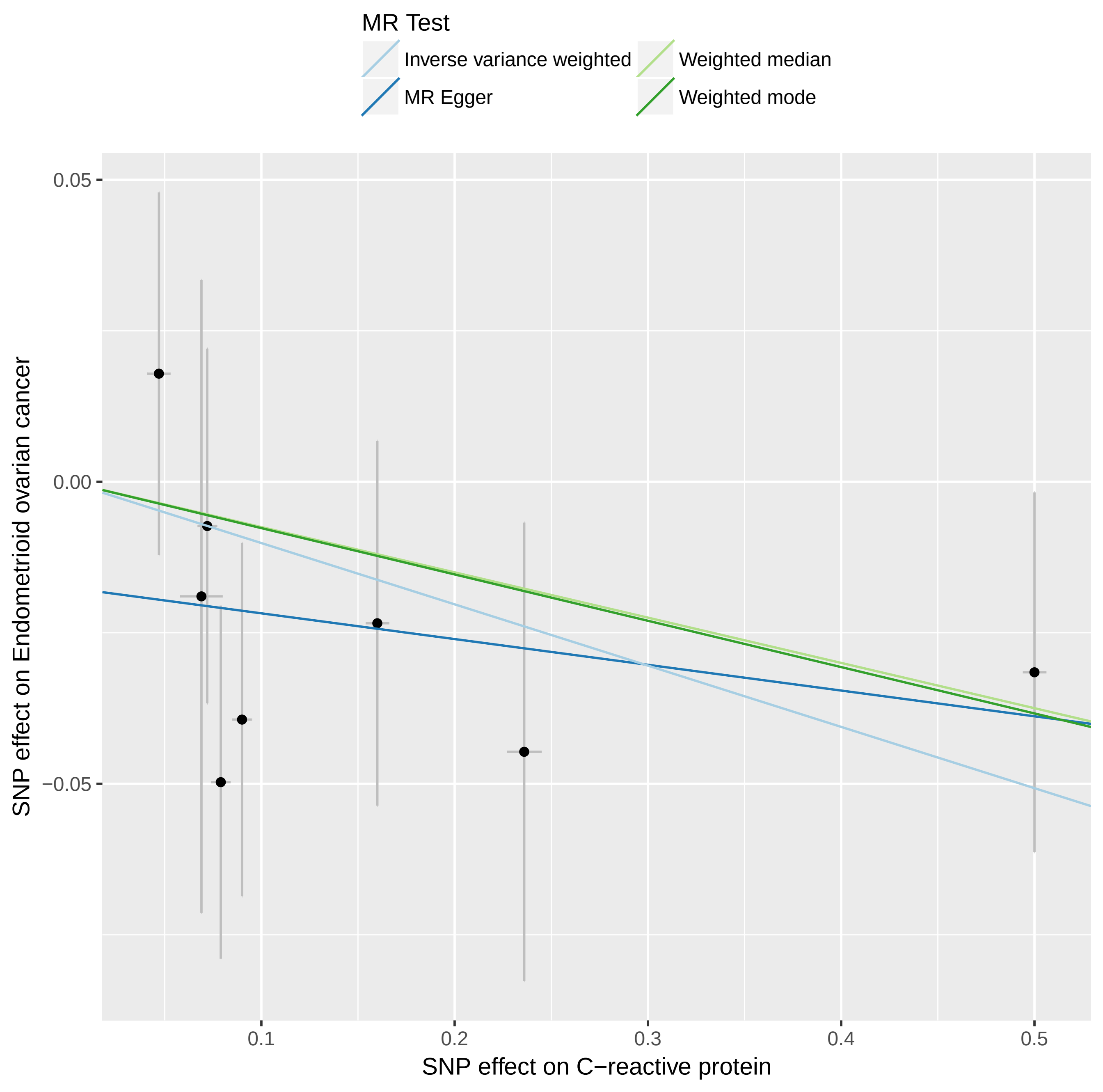

MR Test
Inverse variance weighted
MR Egger
Weighted median
Weighted mode
0.05
0.00
−0.05
0.1
0.2
0.3
0.4
0.5
SNP effect on Endometrioid ovarian cancer
SNP effect on C−reactive protein
