# Supplementary figures and images for: Appraising the role of previously reported risk factors in epithelial ovarian cancer risk: A Mendelian randomization analysis
Source: PLoS Med. 2019 Aug 7;16(8):e1002893. doi: 10.1371/journal.pmed.1002893 (PMC6685606; doi:10.1371/journal.pmed.1002893)

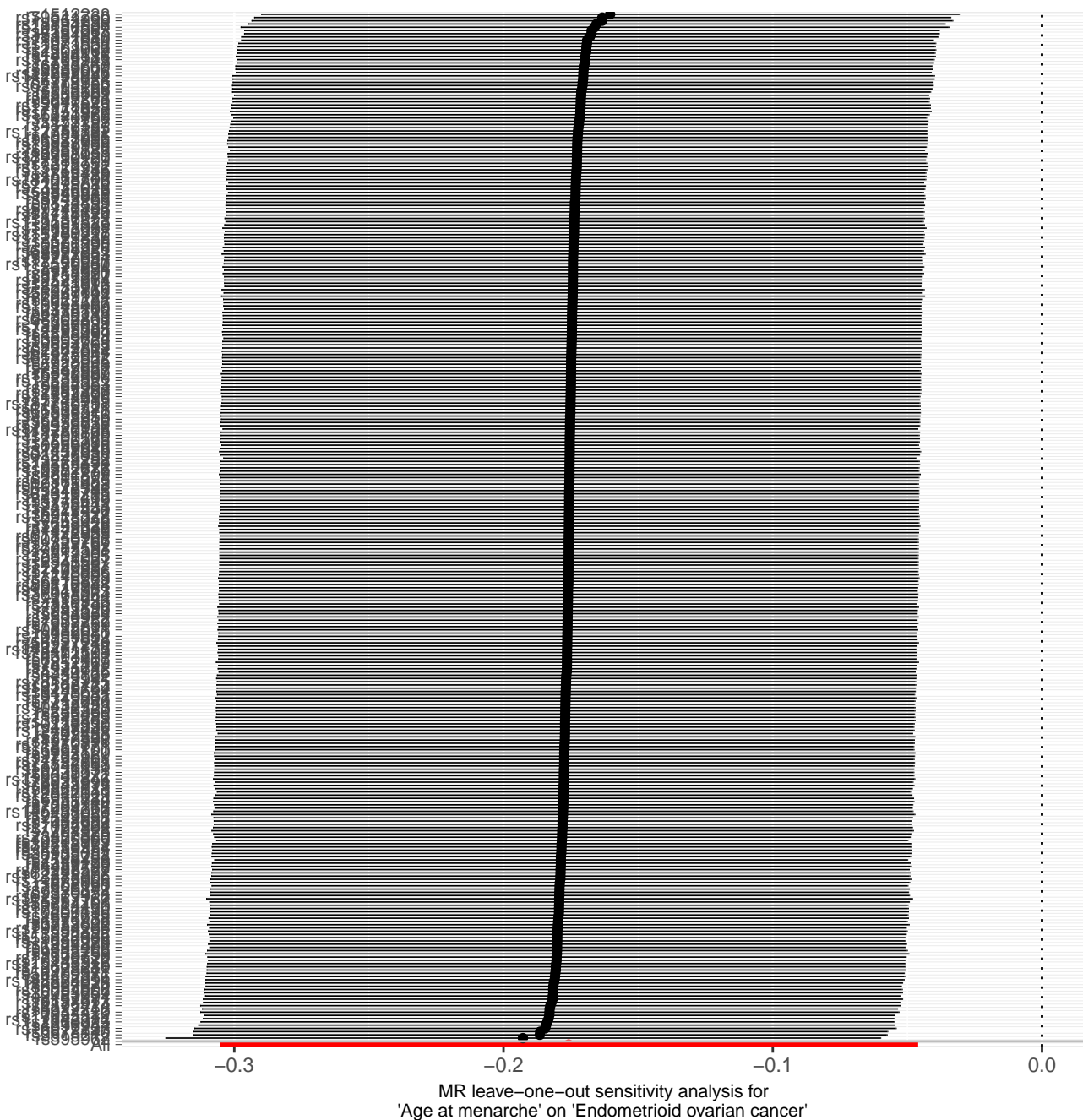

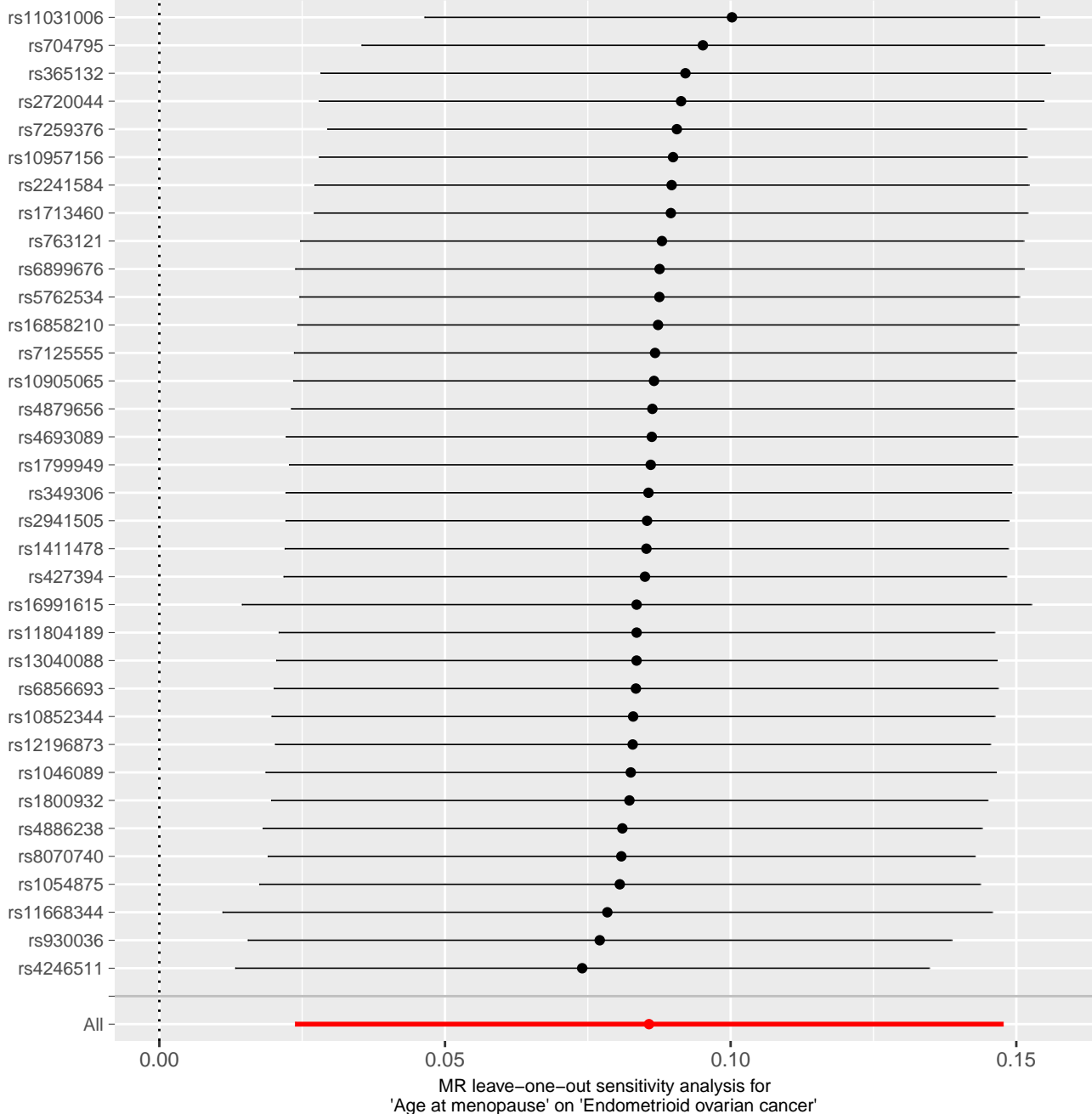

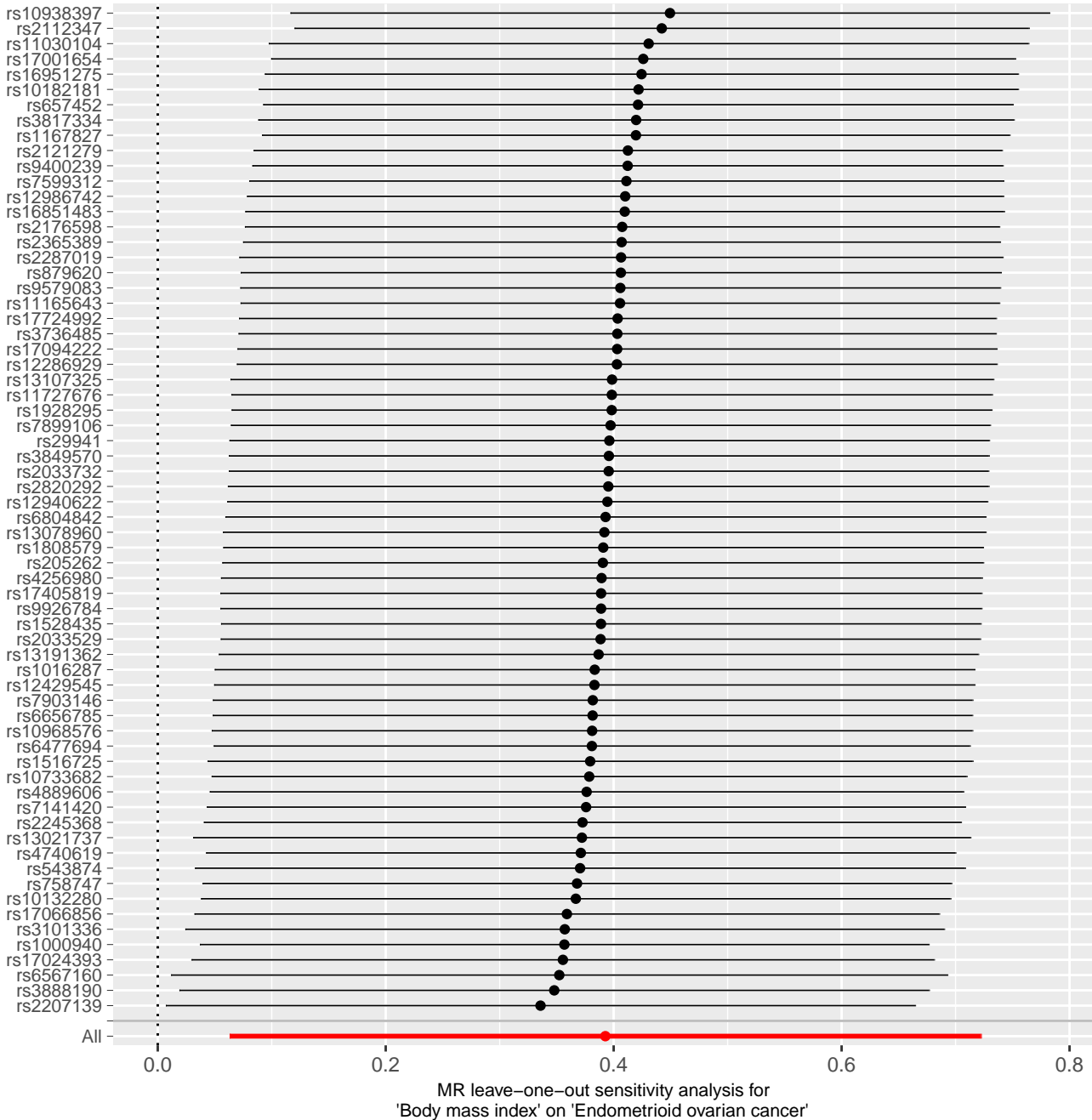

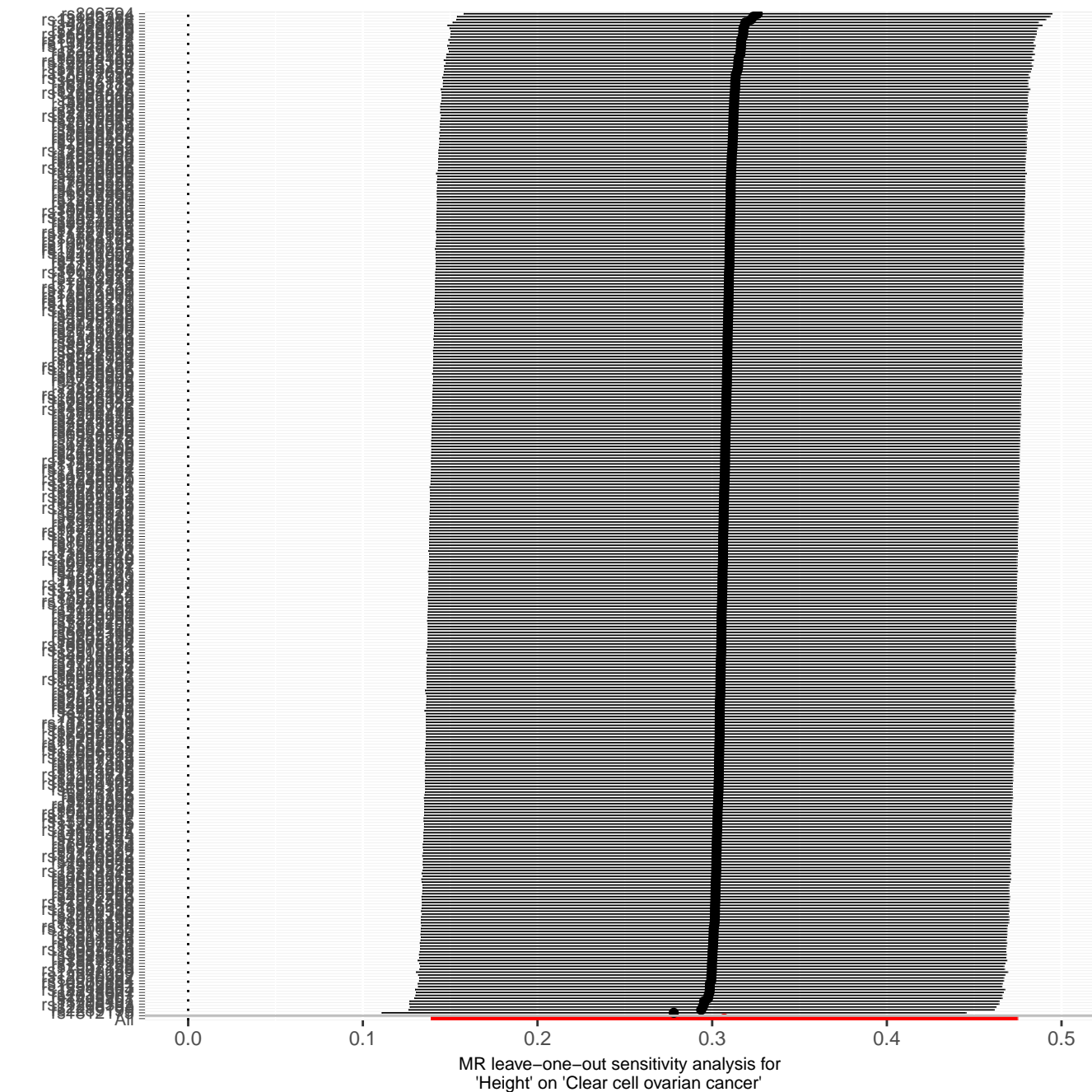

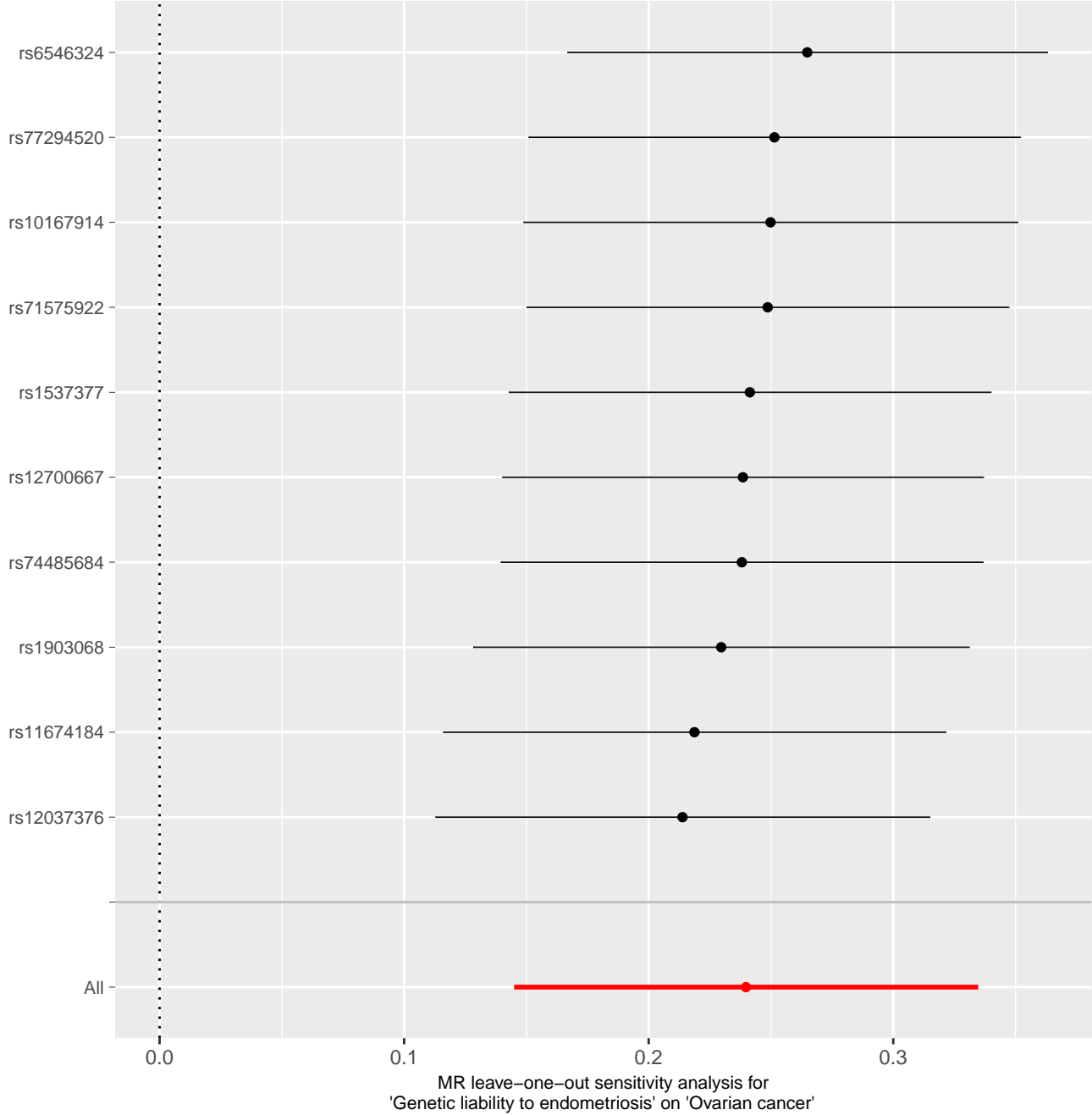

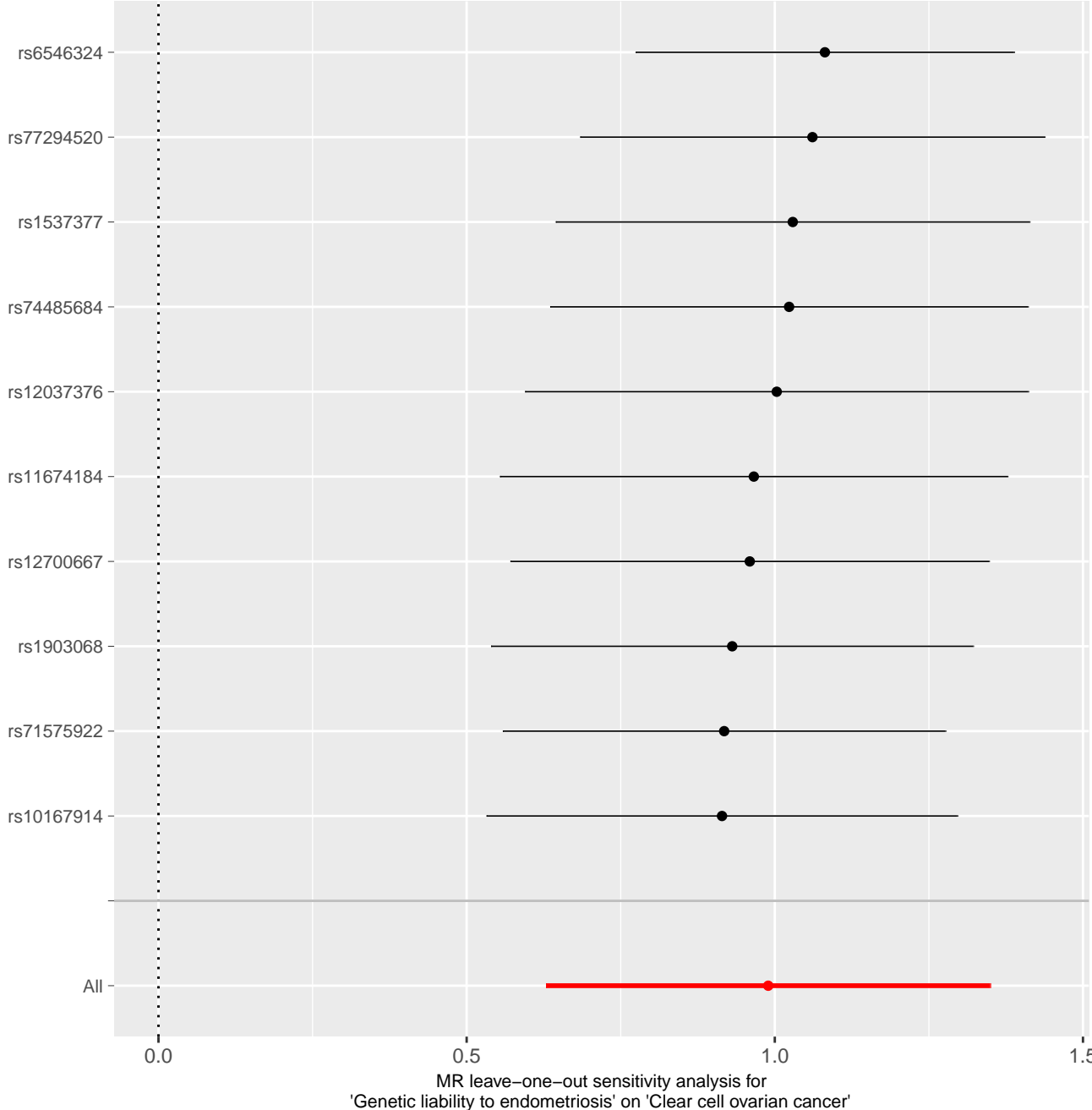

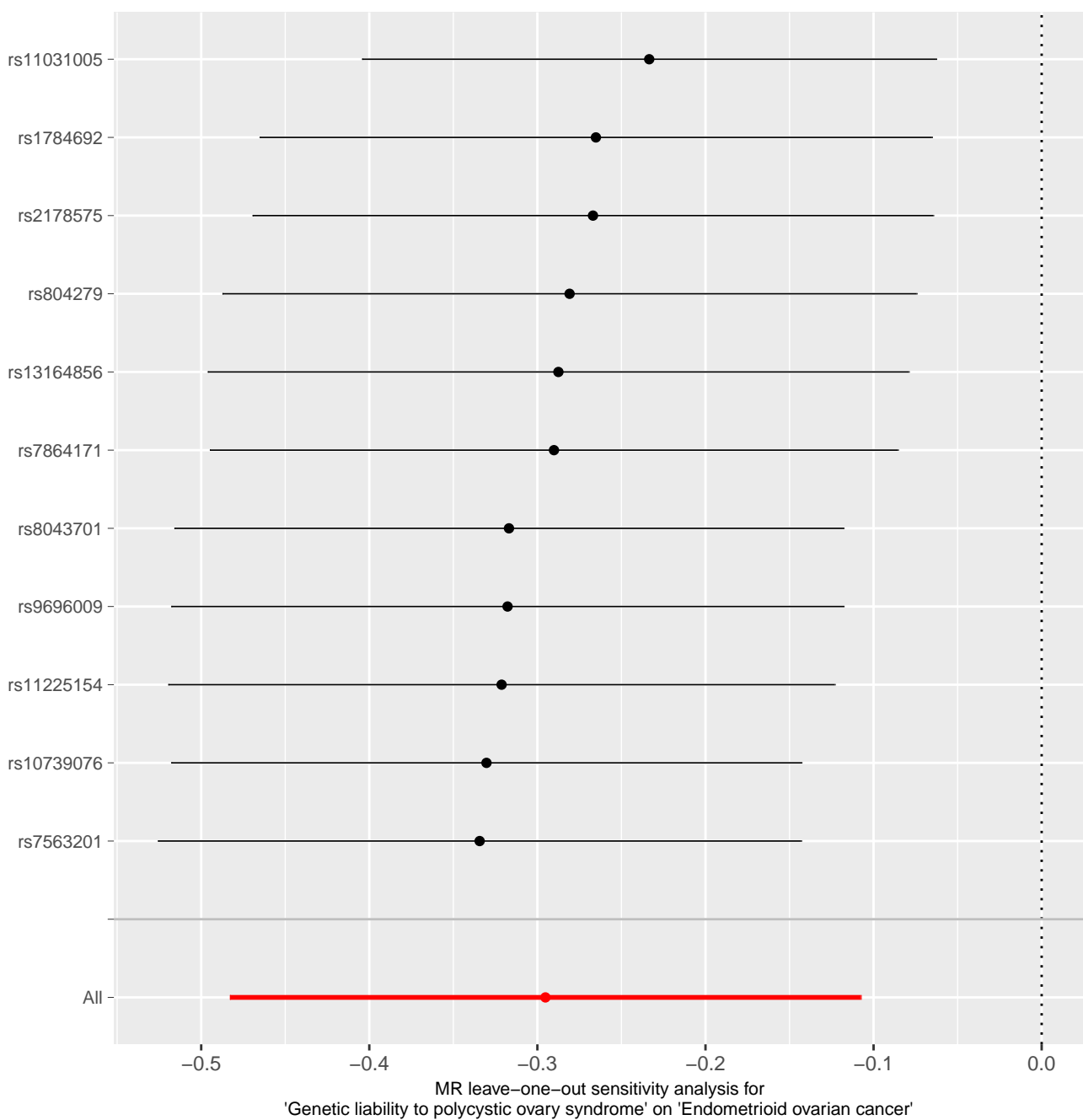

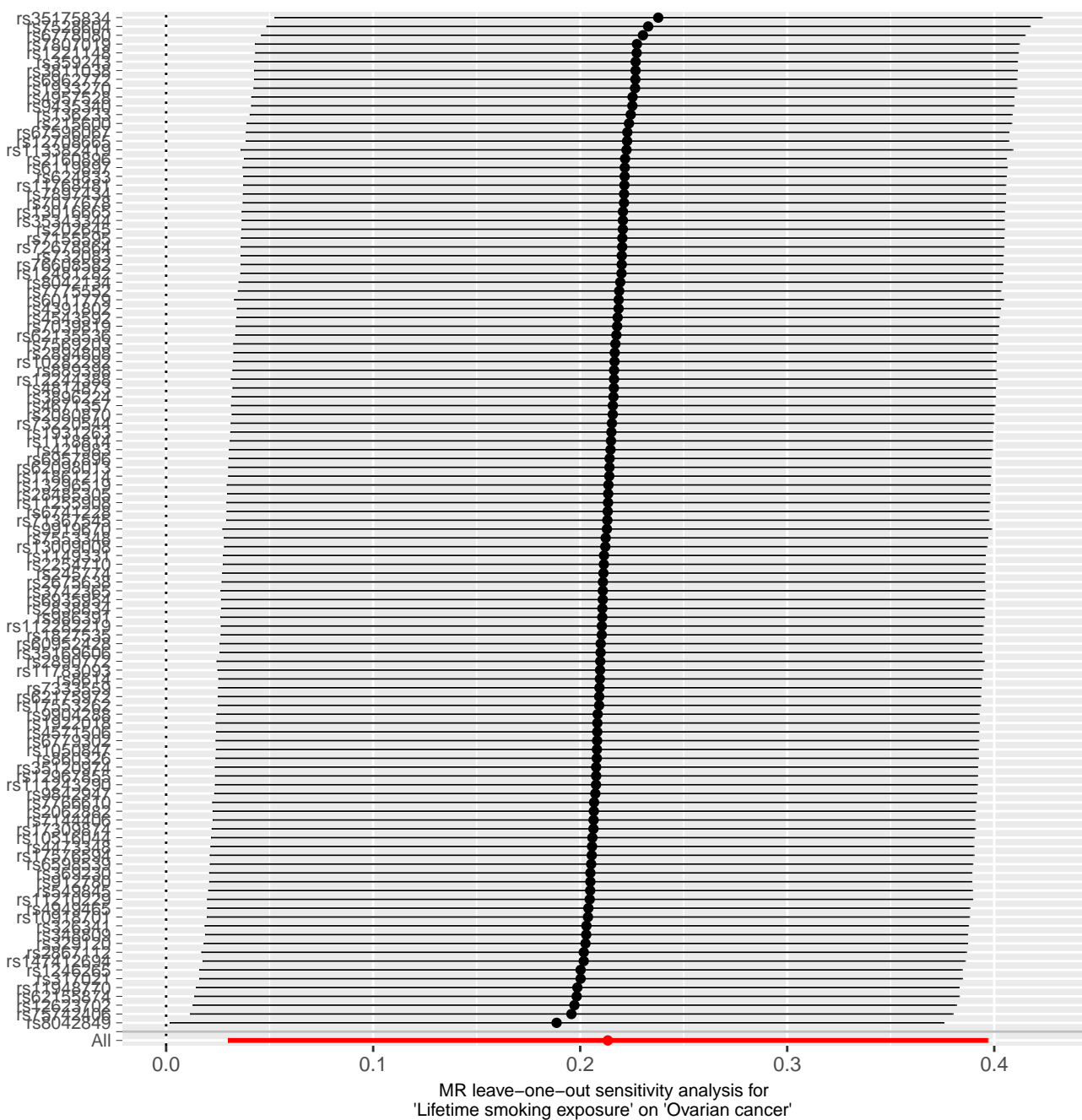

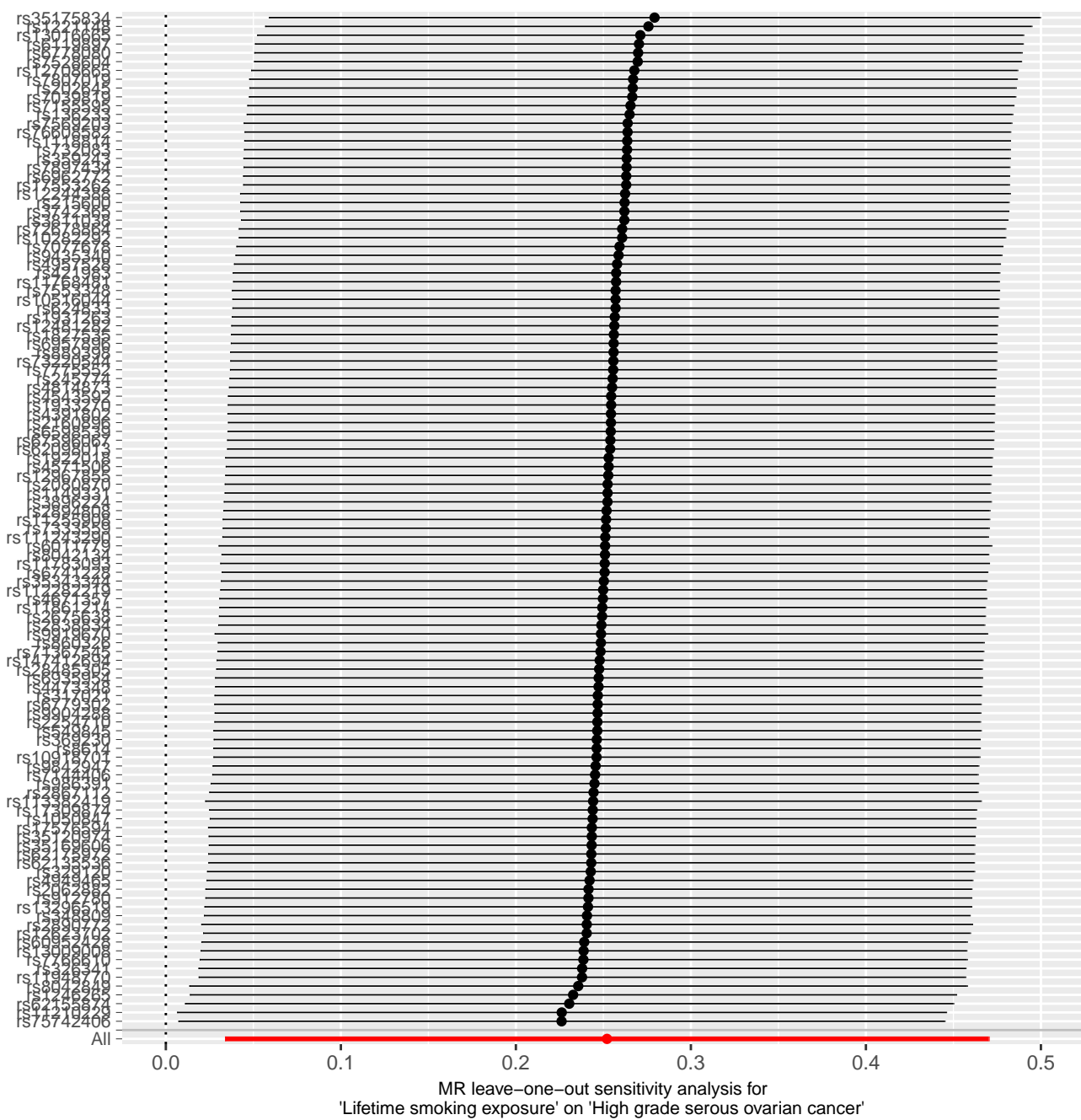

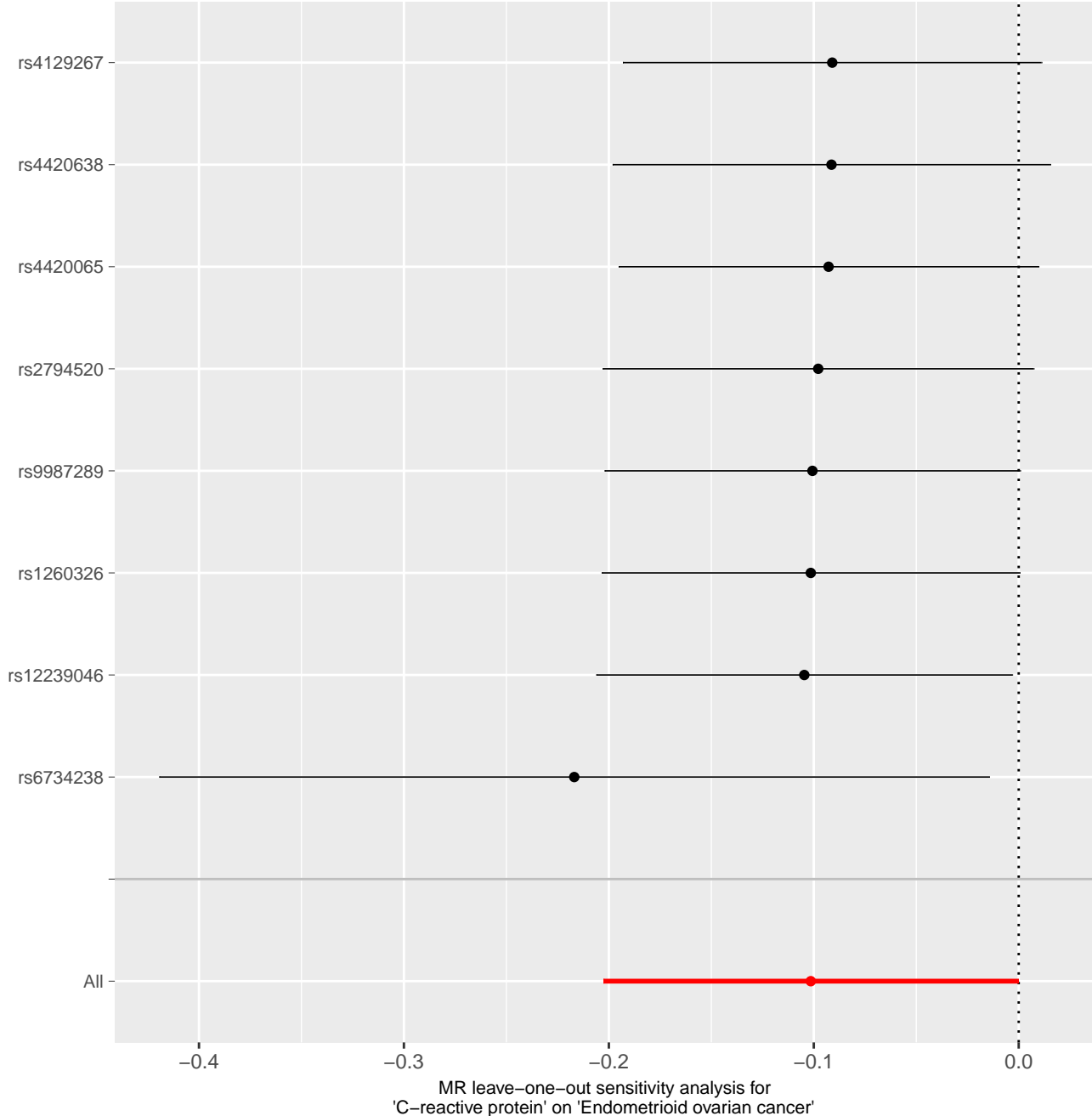

Supplement: S2 Plots — (PDF) [file pmed.1002893.s003.pdf]
