# Supplementary material for: Appraising the role of previously reported risk factors in epithelial ovarian cancer risk: A Mendelian randomization analysis
Source: PLoS Med. 2019 Aug 7;16(8):e1002893. doi: 10.1371/journal.pmed.1002893 (PMC6685606; doi:10.1371/journal.pmed.1002893)
Supplement: S1 Table — (DOCX) [file pmed.1002893.s005.docx]

| **Risk factor** | **Ovarian cancer outcome** | **IVW**  **OR (95% CI)** | ***P*-value** | **MR-Egger regression**  **OR (95% CI)** | ***P*-value** | **MR-Egger intercept**  **OR (95% CI)** | ***P*-value** | **Weighted median**  **OR (95%CI)** | ***P*-value** | **Weighted mode**  **OR (95% CI)** | ***P*-value** |
| --- | --- | --- | --- | --- | --- | --- | --- | --- | --- | --- | --- |
| **Age at menarche** | | | | | | | | | | | |
|  | HGSC | 1.05 (0.97-1.13) | 0.24 | 0.94 (0.82-1.08) | 0.36 | 1.00 (1.00-1.01) | 0.06 | 0.99 (0.88-1.11) | 0.82 | 1.03 (0.29-3.63) | 0.97 |
|  | LGSC | 1.08 (0.86-1.36) | 0.50 | 0.93 (0.61-1.41) | 0.73 | 1.01 (0.99-1.02) | 0.39 | 1.03 (0.72-1.46) | 0.88 | 1.33 (2.5e^-5^-7.2e^4^) | 0.38 |
|  | Mucinous | 1.13 (0.95-1.35) | 0.18 | 1.31 (0.95-1.81) | 0.10 | 0.99 (0.98-1.00) | 0.29 | 1.18 (0.89-1.56) | 0.26 | 0.74 (9.5e^-6^-5.9e^4^) | 0.96 |
|  | Endometrioid | 1.19 (1.05-1.36) | 0.008 | 1.17 (0.93-1.48) | 0.19 | 1.00 (0.99-1.01) | 0.86 | 1.15 (0.94-1.41) | 0.17 | 1.26 (0.12-12.8) | 0.85 |
|  | Clear cell | 1.04 (0.87-1.25) | 0.66 | 0.91 (0.71-1.37) | 0.94 | 1.00 (0.99-1.01) | 0.70 | 0.94 (0.69-1.27) | 0.68 | 0.93 (0.02-35.6) | 0.97 |
|  | LMP | 1.06 (0.94-1.21) | 0.35 | 0.93 (0.74-1.17) | 0.55 | 1.00 (1.00-1.01) | 0.18 | 1.05 (0.86-1.28) | 0.66 | 1.02 (0.09-12.0) | 0.99 |
| **Age at natural menopause** | | | | | | | | | | | |
|  | HGSC | 1.02 (0.99-1.05) | 0.31 | 1.03 (0.97-1.11) | 0.35 | 1.00 (0.98-1.01) | 0.58 | 1.02 (0.98-1.07) | 0.39 | 1.02 (0.97-1.08) | 0.36 |
|  | LGSC | 1.01 (0.92-1.12) | 0.77 | 1.10 (0.89-1.36) | 0.36 | 0.98 (0.94-1.02) | 0.39 | 1.08 (0.95-1.23) | 0.26 | 1.07 (0.92-1.26) | 0.38 |
|  | Mucinous | 1.00 (0.92-1.09) | 0.97 | 1.00 (0.82-1.23) | 0.99 | 1.00 (0.96-1.04) | 0.98 | 1.03 (0.92-1.15) | 0.58 | 1.02 (0.90-1.15) | 0.81 |
|  | Endometrioid | 1.09 (1.02-1.16) | 0.007 | 1.13 (0.98-1.30) | 0.10 | 0.99 (0.97-1.02) | 0.57 | 1.11 (1.02-1.20) | 0.01 | 1.11 (1.02-1.21) | 0.03 |
|  | Clear cell | 1.05 (0.96-1.14) | 0.29 | 1.26 (1.05-1.52) | 0.02 | 1.00 (0.96-1.04) | 0.03 | 1.11 (0.99-1.25) | 0.08 | 1.16 (1.02-1.31) | 0.03 |
|  | LMP | 1.04 (0.98-1.10) | 0.21 | 1.11 (0.98-1.25) | 0.12 | 0.99 (0.96-1.01) | 0.26 | 1.08 (0.99-1.17) | 0.07 | 1.10 (1.00-1.20) | 0.05 |
| **Parity** |  |  |  |  |  |  |  |  |  |  |  |
|  | HGSC | 0.90 (0.30-2.72) | 0.85 | - | - | - | - | - | - | - | - |
|  | LGSC | 0.74 (0.03-21.7) | 0.86 | - | - | - | - | - | - | - | - |
|  | Mucinous | 7.65(0.47-124.8) | 0.15 | - | - | - | - | - | - | - | - |
|  | Endometrioid | 0.19 (0.01-3.23) | 0.25 | - | - | - | - | - | - | - | - |
|  | Clear cell | 0.15 (0.02-1.11) | 0.06 | - | - | - | - | - | - | - | - |
|  | LMP | 1.89 (0.26-14.0) | 0.53 |  |  |  |  |  |  |  |  |

**Supplementary Table 1. IVW and sensitivity analysis estimates for the association of reproductive factors with risk of invasive epithelial ovarian cancer histotypes and low malignant potential tumours**

Estimates are scaled to represent the association of a one-year decrease with age at onset for menarche, a one-year increase in age at onset for natural menopause, and a one-child increase in number of children ever born. IVW = Inverse-variance weighted, HGSC = High grade serous carcinoma, LGSC = Low grade serous carcinoma, LMP = Low malignant potential tumours.
