# Supplementary material for: Appraising the role of previously reported risk factors in epithelial ovarian cancer risk: A Mendelian randomization analysis
Source: PLoS Med. 2019 Aug 7;16(8):e1002893. doi: 10.1371/journal.pmed.1002893 (PMC6685606; doi:10.1371/journal.pmed.1002893)
Supplement: S2 Table — (DOCX) [file pmed.1002893.s006.docx]

| **Risk factor** | **Ovarian cancer outcome** | **IVW**  **OR (95% CI)** | ***P*-value** | **MR-Egger regression**  **OR (95% CI)** | ***P*-value** | **MR-Egger intercept**  **OR (95% CI)** | ***P*-value** | **Weighted median**  **OR (95%CI)** | ***P*-value** | **Weighted mode**  **OR (95% CI)** | ***P*-value** |
| --- | --- | --- | --- | --- | --- | --- | --- | --- | --- | --- | --- |
| **Body mass index** | | | | | | | | | | | |
|  | HGSC | 1.26 (1.06-1.50) | 0.01 | 1.05 (0.63-1.75) | 0.85 | 1.01 (0.99-1.02) | 0.47 | 1.17 (0.91-1.50) | 0.21 | 0.95 (0.53-1.35) | 0.49 |
|  | LGSC | 1.27 (0.73-2.20) | 0.40 | 3.35 (0.68-16.6) | 0.14 | 0.97 (0.93-1.02) | 0.21 | 1.51 (0.71-3.22) | 0.28 | 1.46 (0.45-4.79) | 0.53 |
|  | Mucinous | 1.15 (0.73-1.79) | 0.55 | 4.06 (1.15-14.4) | 0.03 | 0.96 (0.93-1.00) | 0.04 | 1.21 (0.65-2.24) | 0.55 | 1.23 (0.44-3.48) | 0.69 |
|  | Endometrioid | 1.48 (1.07-2.06) | 0.02 | 2.31 (0.89-6.00) | 0.09 | 0.99 (0.96-1.01) | 0.34 | 1.98 (1.23-3.19) | 0.005 | 2.38 (0.86-6.61) | 0.10 |
|  | Clear cell | 0.83 (0.55-1.26) | 0.39 | 1.41 (0.42-4.65) | 0.58 | 0.99 (0.95-1.02) | 0.37 | 1.36 (0.74-2.50) | 0.33 | 1.82 (0.58-5.70) | 0.31 |
|  | LMP | 1.39 (1.04-1.85) | 0.03 | 1.38 (0.60-3.20) | 0.45 | 1.00 (0.98-1.02) | 0.99 | 1.29 (0.83-1.99) | 0.25 | 1.17 (0.55-2.49) | 0.68 |
| **Height** | | | | | | | | | | | |
|  | HGSC | 1.00 (0.94-1.08) | 0.93 | 1.11 (0.92-1.35) | 0.26 | 1.00 (0.99-1.00) | 0.24 | 0.93 (0.84-1.03) | 0.17 | 0.91 (0.74-1.14) | 0.42 |
|  | LGSC | 0.90 (0.74-1.09) | 0.27 | 1.06 (0.63-1.79) | 0.83 | 0.99 (0.98-1.01) | 0.50 | 0.87 (0.64-1.17) | 0.35 | 0.74 (0.40-1.37) | 0.33 |
|  | Mucinous | 0.99 (0.85-1.17) | 0.94 | 1.10 (0.71-1.69) | 0.67 | 1.00 (0.98-1.01) | 0.63 | 1.03 (0.81-1.31) | 0.79 | 1.12 (0.69-1.83) | 0.65 |
|  | Endometrioid | 1.01 (0.90-1.14) | 0.87 | 0.88 (0.64-1.22) | 0.45 | 1.00 (0.99-1.01) | 0.38 | 1.01 (0.83-1.22) | 0.95 | 1.01 (0.66-1.55) | 0.96 |
|  | Clear cell | 1.36 (1.15-1.61) | 0.0003 | 1.20 (0.76-1.88) | 0.44 | 1.00 (0.99-1.02) | 0.55 | 1.38 (1.07-1.79) | 0.01 | 1.31 (0.72-2.41) | 0.38 |
|  | LMP | 1.09 (0.97-1.22) | 0.15 | 1.01 (1.00-1.02) | 0.20 | 0.90 (0.66-1.22) | 0.50 | 1.09 (0.92-1.30) | 0.32 | 1.27 (0.85-1.89) | 0.24 |

**Supplementary Table 2. IVW and sensitivity analysis estimates for the association of anthropometric traits with risk of invasive epithelial ovarian cancer histotypes and low malignant potential tumours**

Estimates are scaled to represent the association of a one-SD increase in body mass index (kg/m^2^) and a one-SD increase in height (cm). IVW = Inverse-variance weighted, HGSC = High grade serous carcinoma, LGSC = Low grade serous carcinoma, LMP = Low malignant potential tumours.
