# Supplementary material for: Appraising the role of previously reported risk factors in epithelial ovarian cancer risk: A Mendelian randomization analysis
Source: PLoS Med. 2019 Aug 7;16(8):e1002893. doi: 10.1371/journal.pmed.1002893 (PMC6685606; doi:10.1371/journal.pmed.1002893)
Supplement: S3 Table — (DOCX) [file pmed.1002893.s007.docx]

**Supplementary Table 3. IVW and sensitivity analysis estimates for the association of clinical factors with risk of invasive epithelial ovarian cancer histotypes and low malignant potential tumours**

| **Risk factor** | **Ovarian cancer outcome** | **IVW**  **OR (95% CI)** | ***P*-value** | **MR-Egger regression**  **OR (95% CI)** | ***P*-value** | **MR-Egger intercept**  **OR (95% CI)** | ***P*-value** | **Weighted median**  **OR (95%C)** | ***P*-value** | **Weighted mode**  **OR (95% CI)** | ***P*-value** |
| --- | --- | --- | --- | --- | --- | --- | --- | --- | --- | --- | --- |
| **Genetic liability to endometriosis** | | | | | | | | | | | |
|  | HGSC | 1.07 (1.02-1.12) | 0.007 | 1.26 (0.99-1.60) | 0.10 | 0.98 (0.96-1.01) | 0.21 | 1.02 (0.93-1.12) | 0.70 | 0.99 (0.75-1.31) | 0.95 |
|  | LGSC | 1.10 (0.95-1.27) | 0.22 | 1.33 (0.59-2.99) | 0.51 | 0.98 (0.90-1.07) | 0.65 | 1.00 (0.67-1.49) | 0.99 | 1.00 (0.69-1.45) | 0.99 |
|  | Mucinous | 1.12 (0.98-1.28) | 0.11 | 1.05 (0.49-2.26) | 0.91 | 1.01 (0.93-1.09) | 0.87 | 1.01 (0.92-1.11) | 0.78 | 1.01 (0.73-1.38) | 0.96 |
|  | Endometrioid | 1.14 (1.04-1.24) | 0.004 | 0.95 (0.60-1.52) | 0.84 | 1.02 (0.97-1.07) | 0.48 | 1.01 (0.95-1.07) | 0.73 | 0.95 (0.41-2.17) | 0.90 |
|  | Clear cell | 1.49 (1.29-1.73) | 7.4e^-8^ | 1.82 (0.81-4.07) | 0.18 | 0.98 (0.90-1.07) | 0.64 | 1.20 (1.00-1.43) | 0.05 | 1.21 (0.20-7.24) | 0.84 |
|  | LMP | 1.12 (1.04-1.22) | 0.006 | 1.05 (0.68-1.62) | 0.83 | 1.01 (0.96-1.05) | 0.76 | 1.09 (1.03-1.15) | 0.003 | 1.09 (0.46-2.56) | 0.85 |
| **Genetic liability to polycystic ovary syndrome** | | | | | | | | | | | |
|  | HGSC | 0.99 (0.94-1.04) | 0.69 | 1.00 (0.76-1.30) | 0.97 | 0.98 (0.96-1.01) | 0.21 | 0.98 (0.93-1.04) | 0.53 | 0.99 (0.91-1.07) | 0.73 |
|  | LGSC | 1.13 (1.00-1.25) | 0.04 | 1.00 (0.59-1.69) | 0.99 | 0.98 (0.90-1.07) | 0.65 | 1.14 (0.99-1.32) | 0.07 | 1.12 (0.90-1.40) | 0.33 |
|  | Mucinous | 1.06 (0.97-1.16) | 0.22 | 0.81 (0.52-1.25) | 0.36 | 1.01 (0.93-1.09) | 0.87 | 1.06 (0.93-1.20) | 0.38 | 1.06 (0.84-1.35) | 0.62 |
|  | Endometrioid | 0.89 (0.82-0.96) | 0.002 | 0.77 (0.53-1.12) | 0.20 | 1.02 (0.97-1.07) | 0.48 | 0.87 (0.78-0.95) | 0.003 | 0.84 (0.71-0.99) | 0.07 |
|  | Clear cell | 1.01 (0.91-1.12) | 0.82 | 0.88 (0.53-1.46) | 0.64 | 0.98 (0.90-1.07) | 0.64 | 1.00 (0.88-1.14) | 0.97 | 0.93 (0.75-1.15) | 0.52 |
|  | LMP | 1.03 (0.97-1.10) | 0.37 | 1.01 (0.74-1.37) | 0.95 | 1.00 (0.96-1.04) | 0.90 | 1.02 (0.94-1.11) | 0.60 | 1.02 (0.89-1.17) | 0.77 |
| **Genetic liability to type 2 diabetes** | | | | | | | | | | | |
|  | HGSC | 0.98 (0.94-1.02) | 0.36 | 0.98 (0.76-1.26) | 0.83 | 1.00 (0.97-1.04) | 0.99 | 0.98 (0.93-1.02) | 0.28 | 0.96 (0.88-1.05) | 0.38 |
|  | LGSC | 0.98 (0.89-1.08) | 0.63 | 0.92 (0.54-1.58) | 0.77 | 1.01 (0.94-1.08) | 0.83 | 0.97 (0.84-1.10) | 0.60 | 0.94 (0.76-1.15) | 0.54 |
|  | Mucinous | 0.98 (0.91-1.07) | 0.70 | 0.82 (0.52-1.28) | 0.40 | 1.03 (0.97-1.09) | 0.43 | 0.98 (0.89-1.09) | 0.74 | 0.98 (0.84-1.14) | 0.78 |
|  | Endometrioid | 1.05 (0.98-1.13) | 0.20 | 0.91 (0.60-1.40) | 0.69 | 1.02 (0.96-1.08) | 0.54 | 1.02 (0.94-1.11) | 0.65 | 1.01 (0.90-1.14) | 0.83 |
|  | Clear cell | 0.99 (0.90-1.07) | 0.75 | 0.80 (0.49-1.31) | 0.41 | 1.03 (0.96-1.10) | 0.43 | 1.00 (0.90-1.12) | 0.95 | 1.02 (0.83-1.25) | 0.83 |
|  | LMP | 1.05 (0.99-1.12) | 0.11 | 1.11 (0.76-1.62) | 0.61 | 0.99 (0.94-1.05) | 0.80 | 1.05 (0.97-1.13) | 0.25 | 1.05 (0.91-1.20) | 0.53 |

Estimates are scaled to represent the association of a 50% higher odds liability to endometriosis, polycystic ovary syndrome, or type 2 diabetes. IVW = Inverse-variance weighted, HGSC = High grade serous carcinoma, LGSC = Low grade serous carcinoma, LMP = Low malignant potential tumours.
