# Supplementary material for: Appraising the role of previously reported risk factors in epithelial ovarian cancer risk: A Mendelian randomization analysis
Source: PLoS Med. 2019 Aug 7;16(8):e1002893. doi: 10.1371/journal.pmed.1002893 (PMC6685606; doi:10.1371/journal.pmed.1002893)
Supplement: S4 Table — (DOCX) [file pmed.1002893.s008.docx]

**Supplementary Table 4. IVW and sensitivity analysis estimates for the association of lifestyle factors with risk of invasive epithelial ovarian cancer histotypes and low malignant potential tumours**

| **Risk factor** | **Ovarian cancer outcome** | **IVW**  **OR (95% CI)** | ***P*-value** | **MR-Egger regression**  **OR (95% CI)** | ***P*-value** | **MR-Egger intercept**  **OR (95% CI)** | ***P*-value** | **Weighted median**  **OR (95%C)** | ***P*-value** | **Weighted mode**  **OR (95% CI)** | ***P*-value** |
| --- | --- | --- | --- | --- | --- | --- | --- | --- | --- | --- | --- |
| **Lifetime smoking exposure** | | | | | | | | | | | |
|  | HGSC | 1.44 (1.05-1.98) | 0.02 | 2.67 (0.78-9.16) | 0.12 | 0.99 (0.98-1.01) | 0.31 | 1.65 (1.02-2.67) | 0.04 | 2.15 (0.78-5.89) | 0.14 |
|  | LGSC | 1.04 (0.38-2.88) | 0.94 | 0.06 (0.00-2.90) | 0.16 | 1.03 (0.99-1.07) | 0.14 | 0.69 (0.17-2.87) | 0.61 | 0.19 (0.01-4.54) | 0.31 |
|  | Mucinous | 1.41 (0.63-3.17) | 0.41 | 0.50 (0.02-11.9) | 0.67 | 1.01 (0.98-1.05) | 0.51 | 0.92 (0.27-3.11) | 0.89 | 0.92 (0.08-10.6) | 0.94 |
|  | Endometrioid | 1.69 (0.93-3.08) | 0.08 | 1.09 (0.11-11.2) | 0.94 | 1.00 (0.98-1.03) | 0.70 | 1.95 (0.84-4.55) | 0.12 | 0.67 (0.08-5.70) | 0.71 |
|  | Clear cell | 0.86 (0.38-1.92) | 0.71 | 2.68 (0.11-62.9) | 0.54 | 0.99 (0.96-1.02) | 0.46 | 0.71 (0.22-2.28) | 0.56 | 0.37 (0.01-12.5) | 0.58 |
|  | LMP | 1.25 (0.84-1.85) | 0.26 | 0.85 (0.19-3.81) | 0.83 | 1.01 (0.98-1.03) | 0.60 | 1.39 (0.78-2.47) | 0.27 | 0.98 (0.23-4.15) | 0.97 |

Causal estimates are scaled to represent the association of a one-unit increase in lifetime smoking exposure. IVW = Inverse-variance weighted, HGSC = High grade serous carcinoma, LGSC = Low grade serous carcinoma, LMP = Low malignant potential tumours.
