# Supplementary material for: Appraising the role of previously reported risk factors in epithelial ovarian cancer risk: A Mendelian randomization analysis
Source: PLoS Med. 2019 Aug 7;16(8):e1002893. doi: 10.1371/journal.pmed.1002893 (PMC6685606; doi:10.1371/journal.pmed.1002893)
Supplement: S5 Table — (DOCX) [file pmed.1002893.s009.docx]

**Supplementary Table 5. IVW and sensitivity analysis estimates for the association of molecular risk factors with risk of invasive epithelial ovarian cancer histotypes and low malignant potential tumours**

Causal estimates are scaled to represent the association of a one-unit increase in natural log-transformed CRP (mg/L), a one-unit increase in natural log-transformed sex hormone-binding globulin (nmol/L), and a one-unit increase in natural log 25-hydroxyvitamin D (ng/mL). IVW = Inverse-variance weighted, HGSC = High grade serous carcinoma, LGSC = Low grade serous carcinoma, LMP = Low malignant potential tumours.

| **Risk factor** | **Ovarian cancer outcome** | **IVW**  **OR (95% CI)** | ***P*-value** | **MR-Egger regression**  **OR (95% CI)** | ***P*-value** | **MR-Egger intercept**  **OR (95% CI)** | ***P*-value** | **Weighted median**  **OR (95%CI)** | ***P*-value** | **Weighted mode**  **OR (95% CI)** | ***P*-value** |
| --- | --- | --- | --- | --- | --- | --- | --- | --- | --- | --- | --- |
| **C-reactive protein** | | | | | | | | | | | |
|  | HGSC | 0.99 (0.93-1.05) | 0.66 | 1.03 (0.94-1.11) | 0.58 | 0.99 (0.97-1.01) | 0.26 | 0.99 (0.93-1.05) | 0.65 | 0.99 (0.93-1.06) | 0.86 |
|  | LGSC | 0.89 (0.69-1.14) | 0.36 | 0.79 (0.54-1.15) | 0.27 | 1.04 (0.95-1.12) | 0.43 | 0.88 (0.73-1.05) | 0.15 | 0.88 (0.73-1.06) | 0.21 |
|  | Mucinous | 0.90 (0.78-1.04) | 0.14 | 0.92 (0.74-1.14) | 0.46 | 0.99 (0.95-1.04) | 0.82 | 0.91 (0.78-1.07) | 0.25 | 0.91 (0.77-1.07) | 0.27 |
|  | Endometrioid | 0.90 (0.82-1.00) | 0.049 | 0.96 (0.83-1.11) | 0.59 | 0.99 (0.95-1.04) | 0.82 | 0.93 (0.83-1.03) | 0.18 | 0.93 (0.83-1.03) | 0.20 |
|  | Clear cell | 1.00 (0.87-1.16) | 0.96 | 0.96 (0.78-1.18) | 0.69 | 1.01 (0.97-1.06) | 0.55 | 0.98 (0.84-1.13) | 0.76 | 0.97 (0.83-1.13) | 0.71 |
|  | LMP | 0.99 (0.89-1.11) | 0.86 | 1.01 (0.85-1.20) | 0.93 | 0.99 (0.96-1.03) | 0.79 | 1.01 (0.91-1.13) | 0.81 | 0.99 (0.89-1.10) | 0.86 |
| **Sex hormone-binding globulin** | | | | | | | | | | | |
|  | HGSC | 1.15 (0.89-1.48) | 0.29 | 1.04 (0.62-1.74) | 0.89 | 1.01 (0.98-1.03) | 0.68 | 1.11 (0.82-1.50) | 0.51 | 1.09 (0.78-1.54) | 0.62 |
|  | LGSC | 0.91 (0.29-2.90) | 0.88 | 3.84 (0.44-33.23) | 0.27 | 0.93 (0.84-1.02) | 0.18 | 1.44 (0.58-3.57) | 0.43 | 1.48 (0.63-3.47) | 0.39 |
|  | Mucinous | 1.13 (0.60-2.14) | 0.70 | 0.83 (0.23-3.02) | 0.78 | 1.02 (0.96-1.08) | 0.60 | 1.10 (0.51-2.37) | 0.82 | 0.92 (0.40-2.10) | 0.85 |
|  | Endometrioid | 1.17 (0.59-2.29) | 0.65 | 1.31 (0.30-5.76) | 0.73 | 0.99 (0.93-1.07) | 0.86 | 1.04 (0.58-1.87) | 0.90 | 1.01 (0.52-1.96) | 0.99 |
|  | Clear cell | 1.41 (0.74-2.69) | 0.29 | 3.25 (0.88-12.01) | 0.13 | 0.96 (0.90-1.02) | 0.20 | 1.90 (0.85-4.25) | 0.12 | 1.91 (0.90-4.08) | 0.14 |
|  | LMP | 1.00 (0.58-1.74) | 0.99 | 1.01 (0.30-3.42) | 0.98 | 1.00 (0.94-1.06) | 0.98 | 0.96 (0.56-1.64) | 0.88 | 0.97 (0.52-1.79) | 0.92 |
| **25-hydroxyvitamin D** | |  |  |  |  |  |  |  |  |  |  |
|  | HGSC | 0.98 (0.61-1.56) | 0.92 | 1.68 (0.79-3.59) | 0.27 | 0.97 (0.93-1.01) | 0.20 | 1.09 (0.77-1.54) | 0.63 | 1.14 (0.80-1.61) | 0.51 |
|  | LGSC | 0.64 (0.24-1.75) | 0.39 | 1.62 (0.24-11.1) | 0.66 | 0.95 (0.86-1.04) | 0.35 | 0.71 (0.24-2.11) | 0.54 | 0.76 (0.23-2.46) | 0.67 |
|  | Mucinous | 1.27 (0.56-2.87) | 0.57 | 0.87 (0.18-4.23) | 0.87 | 1.02 (0.95-1.11) | 0.62 | 1.19 (0.50-2.85) | 0.70 | 1.17 (0.45-2.99) | 0.76 |
|  | Endometrioid | 0.83 (0.46-1.50) | 0.54 | 0.99 (0.31-3.11) | 0.98 | 0.99 (0.94-1.05) | 0.76 | 0.92 (0.49-1.72) | 0.78 | 0.93 (0.45-1.89) | 0.84 |
|  | Clear cell | 1.75 (0.77-3.99) | 0.18 | 2.41 (0.49-11.8) | 0.36 | 0.98 (0.91-1.06) | 0.68 | 2.00 (0.85-4.72) | 0.11 | 1.97 (0.77-5.06) | 0.23 |
|  | LMP | 1.02 (0.43-2.42) | 0.96 | 0.39 (0.09-1.62) | 0.28 | 1.06 (0.99-1.14) | 0.22 | 0.85 (0.43-1.68) | 0.65 | 0.75 (0.39-1.47) | 0.45 |
